# Impact of Surface Chemistry and Doping Concentrations on Biofunctionalization of GaN/Ga‒In‒N Quantum Wells

**DOI:** 10.3390/s20154179

**Published:** 2020-07-28

**Authors:** Nilanjon Naskar, Martin F. Schneidereit, Florian Huber, Sabyasachi Chakrabortty, Lothar Veith, Markus Mezger, Lutz Kirste, Theo Fuchs, Thomas Diemant, Tanja Weil, R. Jürgen Behm, Klaus Thonke, Ferdinand Scholz

**Affiliations:** 1 Institute of Inorganic Chemistry I, Ulm University, Albert-Einstein-Allee 11, D-89081 Ulm, Germany; sabyasachi.c@srmap.edu.in (S.C.); or weil@mpip-mainz.mpg.de (T.W.); 2 Institute of Functional Nanosystems, Ulm University, Albert-Einstein-Allee 45, D-89081 Ulm, Germany; Martin.Schneidereit@uni-ulm.de (M.F.S.); Ferdinand.Scholz@uni-ulm.de (F.S.); 3 Institute of Quantum Matter/Semiconductor Physics Group, Ulm University, Albert-Einstein-Allee 45, D-89081 Ulm, Germany; florian.huber@alumni.uni-ulm.de (F.H.); klaus.thonke@uni-ulm.de (K.T.); 4 Department of Chemistry, SRM University AP Andhra Pradesh, Andhra Pradesh 522502, India; 5 Max Planck Institute for Polymer Research, Ackermannweg 10, D-55128 Mainz, Germany; veith@mpip-mainz.mpg.de (L.V.); mezger@mpip-mainz.mpg.de (M.M.); 6 Fraunhofer Institute for Applied Solid State Physics, Tullastrasse 72, D-79108 Freiburg, Germany; Lutz.Kirste@iaf.fraunhofer.de (L.K.); Theodor.Fuchs@iaf.fraunhofer.de (T.F.); 7 Institute of Surface Chemistry and Catalysis, Ulm University, Albert-Einstein-Allee 47, D-89081 Ulm, Germany; thomas.diemant@uni-ulm.de (T.D.); juergen.behm@uni-ulm.de (R.J.B.)

**Keywords:** n-type GaN, p-type GaN, biosensor, chemical functionalization, protein adsorption, self-assembled monolayer

## Abstract

The development of sensitive biosensors, such as gallium nitride (GaN)-based quantum wells, transistors, etc., often makes it necessary to functionalize GaN surfaces with small molecules or even biomolecules, such as proteins. As a first step in surface functionalization, we have investigated silane adsorption, as well as the formation of very thin silane layers. In the next step, the immobilization of the tetrameric protein streptavidin (as well as the attachment of chemically modified iron transport protein ferritin (ferritin-biotin-rhodamine complex)) was realized on these films. The degree of functionalization of the GaN surfaces was determined by fluorescence measurements with fluorescent-labeled proteins; silane film thickness and surface roughness were estimated, and also other surface sensitive techniques were applied. The formation of a monolayer consisting of adsorbed organosilanes was accomplished on Mg-doped GaN surfaces, and also functionalization with proteins was achieved. We found that very high Mg doping reduced the amount of surface functionalized proteins. Most likely, this finding was a consequence of the lower concentration of ionizable Mg atoms in highly Mg-doped layers as a consequence of self-compensation effects. In summary, we could demonstrate the necessity of Mg doping for achieving reasonable bio-functionalization of GaN surfaces.

## 1. Introduction

Gallium nitride (GaN) materials are applied for producing light emitting diodes (LEDs) emitting in the green, blue and ultraviolet spectral range, laser diodes, high power electronic devices and high frequency transistors [1,2,3,4]. In the past decades, there has been an emerging interest in developing GaN-based biosensors for applications in biological, pharmaceutical, or environmental monitoring, such as biomarker detection [5], glucose sensing [6], and for disease monitoring and clinical diagnosis [7]. In comparison to conventional semiconductors, such as silicon [8], GaN was found to be an excellent material for chemical and biological sensing applications, due to its good chemical stability under harsh environmental conditions, its improved thermal stability, attractive optical properties, good electron mobility, fast response time and excellent biocompatibility [9]. Moreover, its piezoelectric properties allow applications in chemically sensitive field-effect transistor structures, which offers opportunities for the detection of various relevant target molecules, such as glucose or DNA [6,10,11,12,13,14].

The covalent attachment of biomolecules on sensor surfaces is often a crucial step for the detection of biomolecules at the inorganic-organic interface [15,16]. An efficient transfer of charges between the inorganic substrate and functionalized organic layers is known to strongly affect the biosensing performance [10,17,18]. This charge transfer is influenced by the compatibility between the energy band gap of the semiconductor and the positions of the HOMO and LUMO energy levels of the bioorganic moieties [10,18]. The wide-bandgap GaN substrate fits well with a range of HOMO-LUMO energy levels of different biomolecules (e.g., DNAs, proteins), making it a promising material for biosensing applications [17,18]. In the past years, considerable efforts have been devoted to the electrical sensing of enzymes [19], antibodies [20], peptides [21], the protein streptavidin [22,23], DNA [24,25] or phosphoric acid derivatives [26] using chemically functionalized GaN. Recently, we have also developed GaInN quantum well (QW) heterostructures as optical biosensors for the detection of the physisorbed iron storage proteins ferritin and apo-ferritin [27,28]. The presence of surface charges introduced by the analyte induces changes in the near-surface band bending, and thus, generates a potential gradient inside the near-surface QW. In this way, a shift in the photoluminescence spectra occurs, which then enables sensing of these biomolecules [27,28].

To build a robust biosensing platform, smaller distances between the immobilized biomolecule and the transducer surface, surface hydrophilicity, and high coverage of the molecular species to be detected need to be achieved [10]. In this regard, surface functionalization of GaN is desirable to attain a good sensor selectivity and to produce suitable adsorption sites for the attachment of linker molecules, such as functionalized silanes. These could form the first organic monolayer(s) that could then capture the desired target biomolecules [10]. Previously, Baur et al. reported the formation of self-assembled monolayers of 3-aminopropyltriethoxysilane (APTES) and octadecyltrimethoxysilane (ODTMS) covalently attached to n-GaN and AlN surfaces [29]. Later, Munoz and coworkers demonstrated the formation of an APTES monolayer on p-GaN and multilayer formation on n-GaN surfaces using angle-resolved X-ray photoelectron spectroscopy (XPS), the latter indicating imperfect surface functionalization on n-GaN surfaces [30]. Both surfaces were functionalized using the same silanization treatment protocol published earlier. Later, Howgate et al. demonstrated the cleavage of self-assembled ODTMS monolayers on n-GaN surfaces upon irradiation with UV light with a wavelength of 254 nm. In contrast, no degradation of the self-assembled monolayers occurred on p-GaN surfaces upon UV irradiation [31]. Moreover, dye functionalized p-GaN surfaces appeared to be highly sensitive to oxygen exposure, in contrast to n-GaN surfaces, where no changes in the emission lifetime of a ruthenium dye were observed, due to the high deactivation efficiency of the dye [32,33]. Until now, mainly specific case studies on GaN functionalization have been reported, and a systematic investigation and understanding of the surface chemistry of differently functionalized sensor surfaces is still elusive, which limits the potential for designing improved GaN-based biosensors.

Therefore, we have analyzed various functionalization strategies of differently doped n-GaN and p-GaN surfaces, including in each case (1) surface hydroxylation after oxidative treatment, (2) silanization based on the two typical silanes APTES and silane-PEG-biotin (SPB), and (3) functionalization with different protein analytes. Microscopic and spectroscopic methods were applied to characterize the obtained surface morphologies and to quantify the extent of chemical functionalization. We observed the formation of a thin APTES layer on p-GaN, whereas already the first oxidation step of n-GaN appeared challenging, limiting all following functionalization events.

SPB-functionalized p-GaN surfaces allowed the attachment of the tetrameric protein streptavidin (Stp), which has a very high affinity for Vitamin H biotin, and which is often used in biological assay systems. A rhodamine-labeled ferritin-biotin complex was prepared and used for direct optical studies of the biomolecular immobilization. We achieved functionalization and organosilane monolayer formation on p-GaN surfaces, and the iron-storage protein ferritin was attached successfully via the biotin-streptavidin recognition event. Our studies contribute to a more systematic understanding of the surface functionalization processes essential for GaN-based biosensor development.

## 2. Materials and Methods

### 2.1. Materials

Biotin-PEG-Silane (MWCO 3400, Laysan Bio Inc., Arab, AL, USA), streptavidin (Promega, Walldorf, Germany), biotin-NHS (98%, Alfa Aesar, Karlsruhe, Germany), APTES (99%, Sigma Aldrich, Darmstadt, Germany), rhodamine B isothiocyanate (Sigma Aldrich, Darmstadt, Germany), sulfuric acid (>95%, Fisher Scientific, Schwerte, Germany), hydrogen peroxide (30%, Merck Millipore, Darmstadt, Germany) and ferritin from equine spleen (Sigma Aldrich, Darmstadt, Germany) were used as purchased without further purification.

### 2.2. Fabrication of GaN and GaInN QW

All GaN-based samples were grown on c-plane oriented sapphire substrates using an Aixtron AIX200/RF metal organic vapor phase epitaxy (MOVPE) reactor. The precursors ammonia (NH_3_), trimethylaluminum (TMAl), trimethylgallium (TMGa), triethylgallium (TEGa), and trimethylindium (TMIn) were transported into the reactor by ultra-pure carrier gases (N_2_ and H_2_). After the growth of a thin AlN nucleation layer, a GaN buffer layer was grown to a thickness of ~1 µm [34]. On top of the buffer layer, in case of QW structures, one or five InGaN quantum wells of 3 nm thickness were grown, separated by 7 nm GaN layers. The final quantum well was subsequently covered by a thin GaN capping layer of 3 nm thickness to enable strong surface charge interaction as a sensor. In order to provide n- or p-type doping within the GaN buffer layers, diluted SiH_4_ or cyclopentadienyl magnesium (Cp_2_Mg) were added as precursors, respectively [27].

### 2.3. Synthesis and Characterization of Protein Conjugates

80 µL ferritin (50 mg/mL) solution was reacted with biotin-N-hydroxysuccinimide (NHS, 6 mg in 100 µL DMF) in 50 mM phosphate buffer (pH 7.4) in a total solution volume of 5 mL. The mixture was stirred overnight at room temperature. The primary amino groups of ferritin of the lysine side chains were conjugated with biotin, which was activated by an NHS-active ester. Afterwards, a Vivaspin 6 centrifugal concentrator (MWCO 20 kDa, VWR) was used to remove the unreacted biotin molecules from the solution. Matrix-assisted laser desorption/ionization time-of-flight (MALDI-ToF) characterization was used to quantify the number of the attached biotin groups to each subunit of ferritin. The increase in molecular mass of ferritin after biotinylation from 20,115 Da (theoretical 19,500 Da) to 20,703 Da confirmed the successful synthesis of the ferritin-biotin complex (yield: 65%) and the attachment of about three biotin groups [35]. Thereafter, the ferritin-biotin complex (300 µL, 2.7 mg/mL) was reacted with rhodamine B isothiocyanate (80 µL, 0.3 mg/mL) in phosphate buffer solution (50 mM, pH 7.4) in a total solution volume of 5 mL and the mixture was stirred for 24 h at room temperature. Ultracentrifugation using Vivaspin 6 (MWCO 20 kDa, VWR) was carried out to purify the ferritin-biotin conjugate from the unreacted molecules. The successful synthesis of the ferritin-biotin-rhodamine complex via an amine-isothiocyanate reaction was confirmed by UV-Vis spectroscopy (Tecan infinite M1000 microplate reader). The rhodamine dye molecules facilitate straight forward optical characterization of successful biofunctionalized surfaces using fluorescence microscopy.

### 2.4. Surface Preparation

After epitaxial growth, the semiconductor samples/structures were cleaned by 5 min ultrasonication, using acetone and isopropanol. Thereafter, the GaN surfaces were subjected to 20 min of piranha treatment in H_2_SO_4_/H_2_O_2_ (3:1 by volume) for surface hydroxylation. Then, the surfaces were dried, and silanization was carried out in 1 mM silane-PEG-biotin solution or 20 mM APTES solution in toluene in an ultrasonic bath for 90 min at a temperature of 50 °C [29]. Afterwards, the oxidized surfaces were ultrasonicated and washed with anhydrous toluene, isopropanol and treated with 3.5 µM (4% vol) acetic acid solution for 30 min to remove multilayer silane networks to facilitate monolayer formation of adsorbed silane molecules. Then, the SPB functionalized surfaces were incubated with streptavidin (200 µL, 20 µg/mL in water) and subsequently with the ferritin-biotin-rhodamine complex (20 µg/mL in water) for 30 min in each reaction step. Finally, thorough milliQ water rinsing was performed to remove any weakly bound molecules.

### 2.5. XPS Characterization

The elemental composition and the chemical state of the elements in the APTES-coated sample surfaces were determined by X-ray Photoelectron Spectroscopy (XPS) measurements using monochromatized Al K_α_ (1486.6 eV) radiation (PHI 5800 MultiTechnique ESCA System, Physical Electronics). A surface spot of 0.8 × 0.8 mm^2^ was used for analysis. The measurements were done at a detection angle of 45°, using pass energies at the analyzer of 93.9 and 29.35 eV for survey and detail spectra, respectively. The samples were neutralized with electrons from a flood gun (current 3 μA) to compensate charging effects. For binding energy calibration, the Ga (3d) peak of the GaN substrate was set to 19.8 eV. The deconvolution of the XPS spectra was carried out using the XPSPEAK4.1 software.

For quantification of the hydroxyl group density in differently doped p-GaN samples, XPS measurements were conducted using a Kratos Axis Ultra^DLD^ spectrometer (Kratos, Manchester, England) with Al K*_α_* radiation. The data were acquired in the hybrid mode, using a 0° take-off angle, defined as the angle between the surface normal and the axis of the analyzer lens. For survey and high-resolution XP spectra, we used analyzer pass energies of 80 eV and 20 eV, respectively. A neutralizer was always used during spectra collection to avoid surface charging effects at the sample surface. The binding energy scale was calibrated to the Au 4f_7/2_ emission at 84.0 eV [36]. For deconvolution of the XPS spectra we used the XPSPEAK4.1 software.

### 2.6. Fluorescence Microscopy

Fluorescence images were taken using a Leica Microsystems AF 6000 LX system equipped with a Leica camera DFC350FXR2. Imaging was done at a resolution of 8 bits and binning 1 × 1. A Leica CY3 filter was used for excitation and emission with a dry 5× PLAN objective. The exposure time was kept at 25 s, the gain at 10.0 and intensity at 5 a.u. The mean fluorescence intensity was evaluated from 33,792 pixels in a rectangular region of interest (ROI). The ImageJ software was used for image analysis.

### 2.7. SIMS Characterization

Time of flight secondary ion mass spectrometry (ToF-SIMS) spectra were collected using an IONTOF TOF.SIMS^5^ NCS instrument (IONTOF GmbH, Münster, Germany) with 30 keV Bi_3_^+^ primary ion pulses at a current of 0.11 pA (cycle time: 150 µs, mass range: 1‒2070 u) on a field of view of 200 × 200 µm^2^, resulting in a primary ion dose density of 1.6 × 10^11^ ions/cm². The ToF-SIMS surface spectra were acquired under static conditions far from the static limit, and the typical information depth can be expected to be within the first few atomic layers of a sample (<1 nm).

In order to determine bulk Mg concentration, some GaN samples were analyzed by dynamic SIMS at the Fraunhofer Institute for Applied Solid State Physics, Freiburg (further on called “dynamic SIMS”), enabling to estimate the doping concentration in other samples grown under similar conditions. In addition, the carrier concentration (electron and hole concentration for n-doped and p-doped samples) was determined by the Van der Pauw method (Hall measurements).

### 2.8. AFM Measurements

Atomic force microscopy (AFM) imaging was performed using a Bruker Dimension FastScan Bio AFM equipped with ScanAsyst mode. The functionalized GaN surface was scanned using line scan rates of 1–2 Hz and 512 samples per line. Measurements were done in the tapping mode in air. AFM images of several areas of the GaN surface were recorded for reproducibility. Images were analyzed by Gwyddion 2.5, and then a Gaussian function was fitted into the height profile to estimate the height and its standard error for each feature.

### 2.9. Contact Angle Measurements

Contact angle measurements were performed on a Data Physics Instruments OCA15 Pro setup. Each sample was subjected to 2–5 µL milliQ water drops deposited by a thin computer-assisted stainless-steel pipette. For each point, a new position on the sample was selected to reduce crosslinking effects between subsequent measurements. Background illumination was provided by a white-LED array which maximized contrast for the detection camera. The evaluation was performed by fitting a straight line to the sample surface and selecting different points on the drop surface to achieve a fitting curve for the droplet shape. The contact angle was then determined by the evaluation software SCA202.

### 2.10. Ellipsometry Measurements

The measurements were performed on a commercial Woollam V-VASE instrument with a spectral range of 0.5–6.5 eV in steps of 10 meV. Every sample was measured at three angles of incidence, namely 50°, 60° and 70°. A parametric (analytical) model function was employed to describe the dielectric functions of sapphire, the AlN nucleation layer, GaN [37], and a surface layer consisting of a Bruggeman effective medium approximation [38] of GaN and voids in a 1:1 ratio. The layer thicknesses of AlN and GaN were estimated by fitting the Fabry-Perot interference fringes in the transparency range of GaN, and the surface layer thickness was determined using the ellipsometric parameters obtained for energies higher than the absorption onset of GaN. While it is not possible to determine the dielectric functions of the surface layer on a complex layer stack, we could determine the relative surface layer thickness differences [39] between different samples with high accuracy. Relative means that if the void fraction used in the Bruggeman approximation was grossly wrong, the absolute surface layer thickness would also be wrong. However, the comparison between different samples of the same type (i.e., all samples within this study) remains possible. In addition, the semiconductor layer thicknesses obtained by ellipsometry coincided well with the MOVPE growth results.

### 2.11. X-Ray Reflectivity

The X-ray reflectivity (XRR) was measured on a 2-circle diffractometer (Rigaku SmartLab) equipped with a 9 kW rotating Cu anode source and parallel beam multilayer optics for monochromatic Cu-Kα radiation (λ = 1.54 nm). Reflected intensities were recorded on a 2-dimensional single photon counting hybrid pixel detector (Rigaku HyPix-3000, 775 × 375 pixels, 100 µm × 100 µm pixel size). Specular reflected X-ray intensities were recorded in the scattering angle range of 0^o^ ≤ 2θ ≤ 10^o^ with 0.01^o^ step width and 60 s counting time per data point. For quantitative analysis using the software Motofit [40], scattering angles were converted to a momentum transfer $q=\frac{4\pi }{\lambda }sin(\theta )$.

## 3. Results and Discussion

**Figure 1:**
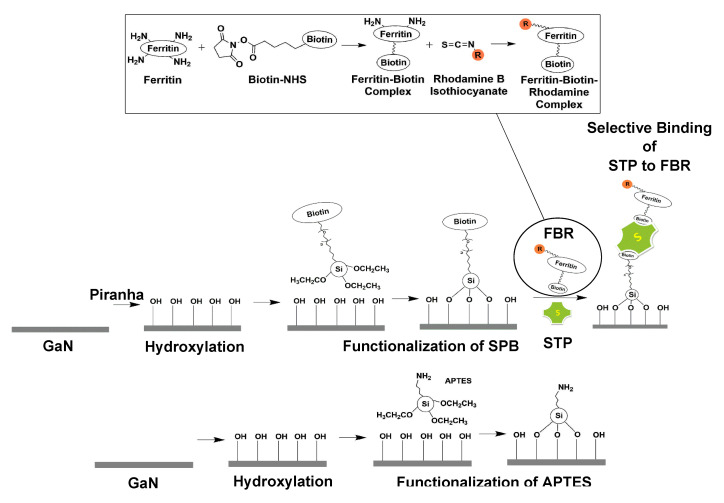
Schematic diagram illustrating the surface biomolecular functionalization routes performed to covalently link two kinds of silane molecules to the gallium nitride (GaN) surfaces, and the resulting protein immobilization. SPB: Silane-PEG-Biotin, STP: Streptavidin, FBR: Ferritin-Biotin-Rhodamine complex, APTES: (3-Aminopropyl) triethoxysilane.

We investigated undoped, n-doped, p-doped GaN and the corresponding QW structures under different conditions (their doping details are summarized in Table 1). First, the surfaces were oxidized via two different methods as described above, either in piranha solution or by oxygen plasma treatment. Thereafter, the oxidized surfaces were exposed to silane-PEG-biotin or APTES solution (Figure 1), wherein the reactive ethoxy groups of the silane undergo hydrolysis to form silanol groups, which could form oligomers in condensation reactions [41]. The hydroxyl (OH) end groups of the silanol species form hydrogen bonds with the surface OH groups, and at elevated temperatures, covalent bonds are formed together with the release of water molecules.

**Figure 2:**
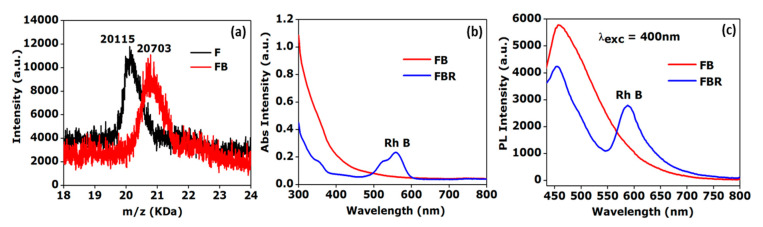
Characterizations of the ferritin-biotin-rhodamine complex in solution: (**a**) Matrix-assisted laser desorption/ionization time-of-flight (MALDI-ToF) spectra of ferritin (black curve, calculated MW—19,500 Da, measured MW—20,115 Da) and of the ferritin-biotin complex subunit (red curve, calculated MW—20,182 Da, measured MW—20,703 Da), (**b**) absorbance spectra, and (**c**) photoluminescence spectra of ferritin-biotin (FB) and the ferritin-biotin-rhodamine (FBR) complex.

The successful synthesis of biotinylated ferritin labelled with rhodamine dyes was evident from (i) the increase in molecular mass of ferritin after biotinylation, which suggested the attachment of an average of three biotin-NHS units per ferritin, and from (ii) the presence of a characteristic peak at 558 nm in the absorbance spectra and the photoluminescence peak at 588 nm when excited with a wavelength of 400 nm (Figure 2). The free biotin end group of biotinylated silane SPB binds non-covalently to one of the four biotin binding pockets of the tetrameric protein streptavidin. As the biotin molecules were only present at the surface, additional biotin binding pockets of streptavidin were still available for binding biotinylated ferritin labeled with rhodamine as depicted in Figure 1.

### 3.1. Oxidation of GaN Surfaces

**Figure 3:**
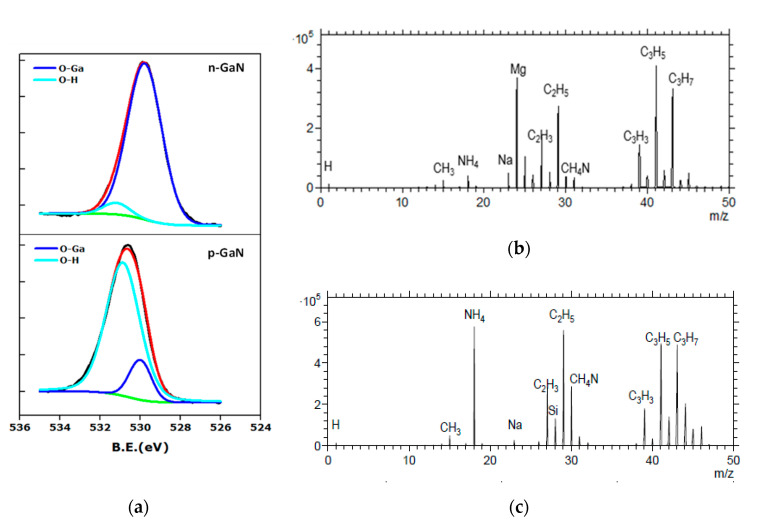
(**a**) O1s XP spectra of hydroxylated n-GaN (top) and p-GaN (bottom) surfaces. Background (green), fitted curve (red), experimental curve (black) and deconvoluted curves corresponding to O‒Ga (dark blue) and O‒H (light blue) bonds, x-axis: Binding energy (eV), y-axis: Intensity (a.u.). Time-of-flight secondary ion mass spectrometry (ToF-SIMS) spectrum of blank (**b**) p-GaN (**A**) and (**c**) n-GaN (**B**) surfaces, x-axis: Mass/charge ratio (m/z), y-axis: Intensity (a.u.).

The aforementioned GaN surfaces were hydroxylated using piranha solution and oxygen plasma treatment at various power settings, such as 100 W, 150 W, and 200 W, and with different irradiation times of 10 min and 20 min for surface oxidation. We have performed XPS measurements to investigate the oxide formation and the OH group density on the hydroxylated GaN surfaces. After piranha treatment of the GaN substrates, the overall intensity of the O1s peak has increased (Figure 3a and Figure S1). This indicates surface oxidation or the formation of an oxide layer. The O1s spectrum for treated p-GaN (**A**) was deconvoluted into peaks at 530 eV and 530.9 eV, which are ascribed to O‒Ga and O‒H species. For the treated n-GaN (**B**), the deconvoluted peaks appeared at 529.8 eV and 531.4 eV with similar assignment [30]. The ratios of the deconvoluted O1s intensities for O‒H and O‒Ga bonds were 0.03 in n-GaN (**B**) and 5.7 in p-GaN (**A**) surfaces, indicating that most of the oxygen uptake resulted in oxide formation (O‒Ga) on n-GaN (**B**), where also much less OH surface groups were formed. In contrast, on p-GaN (**A**), much larger amounts of OH surface groups (in the form of Ga‒O‒H) appeared and relatively little surface oxides were observed [30]. Here, the ratio of the deconvoluted peak areas for O‒H and O‒Ga increased from 3.3 (untreated) to 5.7 (piranha treated) (Figure 3a and Figure S1a), while for n-GaN (**B**) we found no difference in the O1s intensity ratio of the O‒H and O‒Ga species between the untreated and piranha treated samples (Figure S1b). Hence, surface hydroxylation could only be achieved for the p-type sample surface.

To confirm the chemical compositions of the unmodified GaN surfaces, we performed time-of-flight secondary ion mass spectrometry (ToF-SIMS) measurements of the GaN surfaces. These revealed the presence of Si atoms on blank n-GaN (**B**) and of Mg atoms on blank p-GaN (**A**) surface layers (Figure 3b,c). As expected, the silicon atom density on the QW sample (**D**) with the undoped GaN capping layer was much lower than that on n-GaN (**B**) (Figure S2a). The surface images (ion maps) revealed a homogeneous ion signal distribution in the samples (Figure S2b–d). A different type of dopant may lead to a different alignment of the semiconductor energy gap at the surface with the molecular energy gap, which can be a plausible reason for different degree of molecular functionalization at the surface. The piranha solution (H_2_SO_4_/H_2_O_2_) produces reactive hydroxyl radicals that are nucleophilic [30] and could donate electrons to the p-GaN surface, thus facilitating hydroxylation reactions, whereas this is not the case for the surfaces of n-GaN or n-QW [42]. Accordingly, higher coverages of OH groups were generated at the p-type surface as compared to the n-type surfaces.

Furthermore, we investigated the extent of the hydroxylation of the n-GaN (**B**) and p-GaN (**A**) surfaces created by oxygen plasma treatment and the subsequent biomolecule immobilization (Figure 1) by fluorescence spectroscopy of the protein functionalized surfaces. We observed an intense fluorescence on bio-functionalized p-GaN as compared to n-GaN surfaces. The mean fluorescence intensities of blank, 100 W (20 min) and 200 W (10 min) O_2_ plasma treated bio-functionalized n-GaN were measured to be 40 ± 7 a.u., 35 ± 3 a.u. and 49 ± 7 a.u., whereas for p-GaN the mean intensity was found to be 46 ± 4 a.u. (blank) and 64 ± 4 a.u. (treated). The only small increase in mean fluorescence intensity between blank and protein-functionalized n-GaN can be a possible indicator for no significant improvement of the degree of surface hydroxylation of n-type surfaces when using oxygen plasma (Figure S3).

### 3.2. Surface Coating of Oxidized GaN Surfaces with Silanes

Next, we studied the impact of the surface oxidation on the interaction with the silanes APTES and SPB in solution. Piranha treated p-GaN (A), n-GaN (B), u-GaN (C), and u-QW (D) surfaces were functionalized using APTES and SPB using the procedure (Figure 1) described by Baur et al. [29].

**Figure 4:**
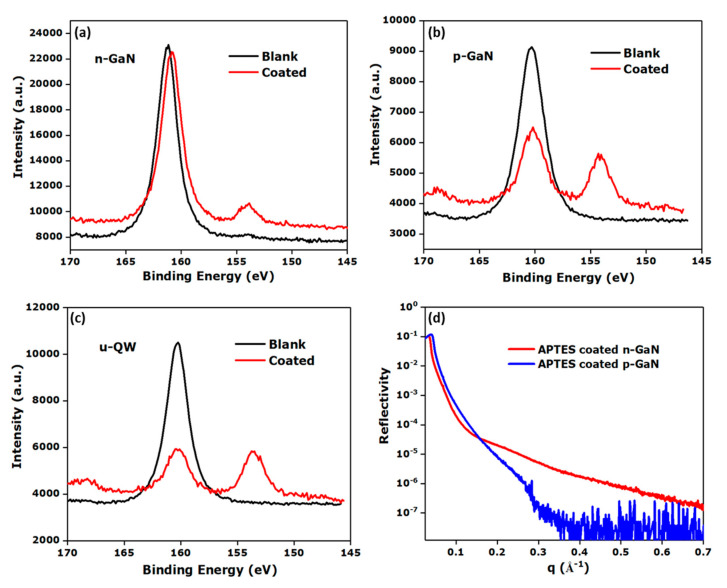
Si (2s) XP spectra of bare and APTES-coated (**a**) Si-doped GaN (n-GaN, **B**), (**b**) Mg-doped GaN (p-GaN, **A**) and (**c**) undoped QW (u-QW, **D**). (**d**) X-ray reflectivity (XRR) pattern of APTES-coated n-GaN (red) (**B**) and p-GaN (blue) (**A**), x-axis: Scattering vector (q).

Surface characterization by XPS was performed to characterize the adsorbed APTES layer on these functionalized surfaces. The Si (2s) peak allowed the detection of Si originating from the adsorbed APTES layer. The signal at around 160 eV (Figure 4) can be ascribed to a Ga (3s) peak, whereas the signal at around 154 eV is assigned to the Si (2s) peak of Si atoms in the APTES molecules [24]. The XPS spectra revealed the presence of functionalized APTES layers on all surfaces (Figure 4a–c and Figure S4).

X-ray reflectivity (XRR) measurements were employed to determine the APTES layer thickness at the surface. For a quantitative analysis of the XRR data, we used a two-layer model consisting of a native oxide layer underneath a layer of organic molecules. The experimental data were fitted using the software Motofit [43]. The resulting parameters obtained by fitting are summarized in Tables S1 and S2. No APTES layer was found on n-GaN surfaces, while a layer with 8.3 Å thickness was detected on p-GaN (Figure 4d) confirming the formation of an APTES monolayer on p-GaN [44]. The XRR pattern recorded from coated n-GaN (Figure 4d) was similar to that obtained on the bare GaN surface [43]. Furthermore, it was observed that the thickness of the APTES layer on p-GaN changed in the following day, presumably due to variations in relative humidity, and thus, adsorption of moisture leading to an increase in thickness to 18.2 Å (Figures S5 and S6b). The values of the surface roughness extracted from the XRR data are in qualitative agreement with the maximum roughness obtained from AFM measurements (Table S1) [43]. In addition, an increase in apparent surface roughness (estimated from an effective medium approximation of the layer thickness) was observed on the APTES-treated u-GaN (**C**) and p-GaN (**A**) surfaces from ellipsometry measurements (Table S3). The presence of the APTES layers can be a plausible reason for the change in surface properties between the blank and treated surfaces [45].

### 3.3. Protein-Functionalization of the GaN Surfaces

**Figure 5:**
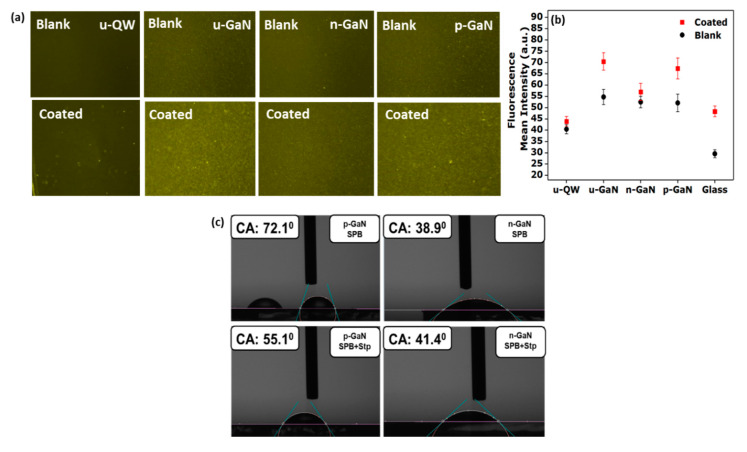
(**a**) Fluorescence images of biomolecule-functionalized undoped QW (u-QW, **D**), undoped GaN (u-GaN, **C**), Si‒doped GaN (n-GaN, **B**) and Mg‒doped GaN (p-GaN, **A**). (**b**) Mean fluorescence intensity (a.u.) of biomolecule coated substrates. (**c**) The contact angle of water drops on bare and silane-PEG-biotin (SPB) coated p-GaN (**A**) and n-GaN (**B**) surfaces with and without streptavidin (Stp).

In the next step, we investigated the functionalization of the p-GaN (**A**), n-GaN (**B**), u-GaN (**C**) and u-QW (**D**) surfaces with the proteins streptavidin and the FBR complex. Even though surface hydroxylation and silanization did not occur at the n-GaN (**B**) surface, it would be interesting to elucidate whether proteins would adsorb weakly on these surfaces, as it is known for many other surfaces, such as gold surfaces [46,47]. In our setup, the biosensor design is based on the non-covalent interactions between the protein streptavidin and biotin, which is known to be among the strongest non-covalent interactions present in nature [48,49]. The resulting biotin-streptavidin complexes are known to be stable against changes in temperature, pH and organic solvents, which make them particularly suitable for designing protein-selective biosensors [48,49]. Silanized GaN surfaces were exposed to streptavidin biomolecules and thereafter incubated with the FBR complex. Between each step, the samples were rinsed with water to remove weakly bound FBR proteins. The protein nanostructures (Streptavidin (Stp) bound FBR complex) immobilized on the surfaces were then imaged by fluorescence microscopy. The observed fluorescence originated from the attached rhodamine molecules. Biofunctionalized p-GaN (**A**) and u-GaN (**C**) surfaces revealed a clearly visible fluorescence signal compared with the glass substrate, whereas the incubation of the proteins on the n-GaN (**B**) surfaces did not result in a fluorescence signal. The differences in the mean fluorescence intensities between the blank and the biofunctionalized surfaces (Figure 5a,b) are summarized in Table 2. The preparation of n-GaN and u-GaN samples is less challenging compared to the p-GaN samples, where parasitic effects have to be considered carefully, such as Mg delay when switching on the Mg source in MOVPE, some memory effects from previous runs or Mg diffusion in the samples, etc. In addition, well-defined p-GaN samples also require additional preparation steps, such as thermal activation of the Mg acceptor to remove hydrogen.

Based on these results the p-GaN (**A**), u-GaN (Y2151, **C**), and glass samples showed larger intensity differences, indicating the presence of protein nanostructures composed of Stp and FBR [50], whereas biofunctionalization of n-GaN (**B**) and u-QW (**D**) was not successful. We have tested several n-doped samples, but protein functionalization of n-GaN and n-QW surfaces was not successful. In addition, functionalized silane layers did not form on the n-doped surfaces from the ellipsometric measurements (Table S3). One could speculate that different surface dopants, such as Si or Mg, generate a different near-surface GaN band bending, which could change the HOMO-LUMO band alignment of the biomolecules. It has been shown that biofunctionalization could be achieved by varying the type of doping impurities [17,18]. Interestingly, we did not observe any weak (unspecific) adsorption of the proteins on the n-GaN and u-QW surfaces, which is an important feature considering their adsorption affinity to other surfaces, such as gold, silver, etc. [46,47]. This is an important feature for achieving protein-selective sensor surfaces, where any weak protein adsorption needs to be suppressed. In addition, measurements of the spatial variation in fluorescence intensity on p-GaN and glass surfaces showed constant intensities from an uncoated region over a step into the coated region of the protein-functionalized substrate (Figure S8a–d) [51] suggesting the formation of a homogeneous layer of the protein complexes on p-GaN (**A**) and u-GaN (**C**) surfaces. The presence of the Si 2s XPS peak at 153 eV, which is related to Si in the APTES molecules in APTES-functionalized surfaces, demonstrated successful surface functionalization for n-GaN (**B**), u-QW (**D**), p-GaN (**A**) (Figure 4a–c) and u-GaN (**C**) (Figure S4). On the other hand, significant fluorescence intensity originating from the protein complexes was only observed for the u-GaN (**C**) and p-GaN (**A**) surfaces (Figure 5a,b). This discrepancy could be explained by an incomplete functionalization of the n-GaN (**B**) and u-QW (**D**) surfaces, with low silane coverages well below a monolayer. These observed changes in fluorescence intensity were also reproduced in other samples (Figures S17–S20). In addition, we observed that at u-GaN (**C** and **G**) surfaces, the protein complexes were attached, whereas they were not bound to u-GaN (**E** and **F**). By ToF-SIMS, we found a slightly higher Mg concentration for u-GaN (**C** and **G**) (180 and 102 a.u.) as compared to other u-GaN samples (<70; Figure S9), which could have a critical influence on the biofunctionalization. However, the relative Mg ion intensity level of p-GaN samples (>10^5^ a.u.) was significantly higher compared to the u-GaN samples. The u-GaN (**G**) sample showed fairly similar Mg surface concentrations as the u-GaN (**C**) sample, which was synthesized from a different MOVPE reactor with changed growth parameters and was grown with a SiN in-situ layer that improved its GaN quality. However, we did not find any relation between such higher GaN quality or the presence or absence of the SiN interlayer and the functionalization potential. For proper comparison, samples (**A**, **B**, **C**, **D**) grown from the same reactor are shown in the results (Figure 5). The reason for the comparably high Mg content within the u-GaN (**C**) sample surface could not be explained yet as no Mg-precursor flow was injected during the growth of the sample. We could only speculate that the low surface Mg concentration in u-GaN samples (**C** and **G**) could shift the Fermi level considerably, so that the near-surface GaN bandgap complements the energy bandgap of protein molecules, thus resulting in successful bio-functionalization in u-GaN samples (**C** and **G**). However, more detailed studies would be required.

Contact angle measurements were performed on the GaN surfaces coated with silane and incubated with and without streptavidin protein (Figure 5c), to assess possible polarity changes of the surfaces. The contact angle of water drops on silane-coated p-GaN (**A**) surface was 67° ± 3°, which decreased to 55° ± 0.5° after streptavidin incubation and concomitant water washing. This reduction is attributed to the presence of a higher number of hydrophilic surface groups (NH_2_, OH, etc.) originating from bound streptavidin [52]. However, in the case of n-GaN (**B**), the measured contact angles for silane-coated surfaces before and after incubation of streptavidin molecules remained essentially constant with 38° ± 0.5° and 43° ± 1°, respectively, indicating the absence of measurable amounts of attached streptavidin in the latter cases (Figure 5c). These observed changes in the contact angle were reproduced in other p-GaN and n-GaN samples (Figures S21 and S22).

### 3.4. Surface Characterization on the Biomolecular Scale

**Figure 6:**
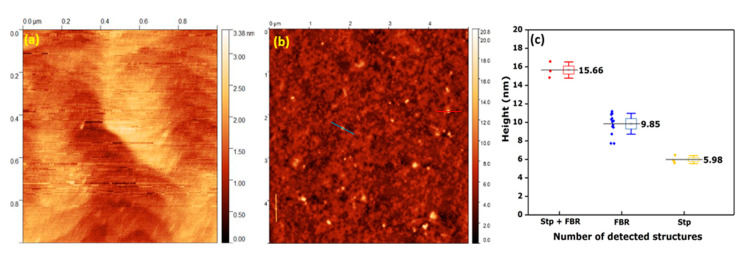
AFM images of (**a**) bare and (**b**) protein functionalized p-GaN (**A**). (**c**) Height distribution of bright features on the coated p-GaN (**A**) surface. Nanostructures were clustered according to their dimensions, and three different clusters were identified with about 6 nm, 9.9 nm and 15.7 nm, which were assigned to Stp (5 nm), FBR complexes (10 nm) and Stp bound FBR complexes (15 nm).

In order to characterize the morphology of the protein-coated p-GaN (**A**) surfaces, we recorded AFM images in the dry state. The smooth wavy structures obtained for the bare surfaces (see Figure 6a) are a typical characteristic of high quality GaN surfaces. For the protein functionalized p-GaN (**A**) surfaces, the AFM images (Figure 6b) showed the presence of many nanosized features of different dimensions with average heights of 16 ± 0.9 nm, 10 ± 1.1 nm and 6 ± 0.4 nm (see Figure 6b,c). Based on the known dimensions of streptavidin and ferritin, with heights of about 5–6 nm and 8–12 nm, respectively, we assigned the nanostructures with heights of about 16 nm to streptavidin bound FBR complexes, whereas the objects with about 10 nm and 6 nm dimensions could be related to weakly bound FBR complexes and streptavidin (Figure S10d,e) [52]. We have observed the same nano-features in AFM images measured on other bio-functionalized p-GaN samples (Figure S23).

### 3.5. Effect of Doping Concentrations on Protein Functionalization

To further improve the surface functionalization of the n-GaN surfaces, the influence of the n-doping level on protein immobilization on n-GaN was studied. Using Si doping levels of <10^17^, 2 × 10^17^ (both nominally undoped) and 9 × 10^18^ cm^–3^, we found no change in the extent of functionalization (Figure S11): All samples remained non-functionalized. In addition, silanization experiments of n-GaN were performed in different solvents, such as THF, water, and toluene, but subsequent protein functionalization was still unsuccessful, as evident from the small differences in the mean fluorescence intensity between blank and treated samples.

**Figure 7:**
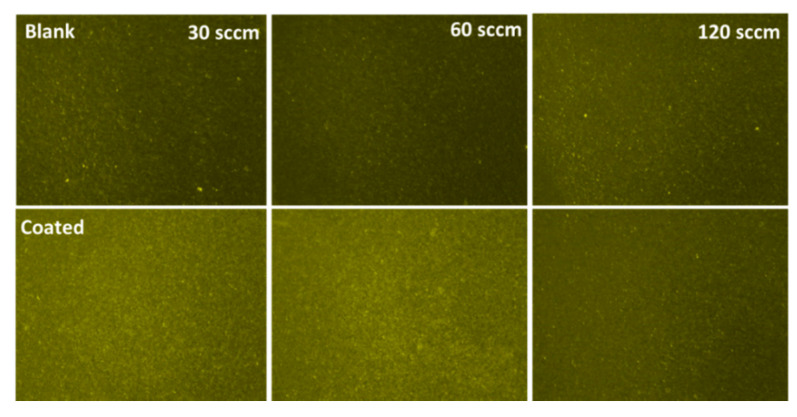
Fluorescence images of functionalized p-GaN surfaces with Cp_2_Mg injection levels of 30 sccm, 60 sccm, and 120 sccm.

In the same way, the effect of p-type doping at various doping levels on the surface functionalization of the as-prepared p-GaN was studied to identify the regime of successful molecular functionalization of low electron p-type surfaces as reported by Arranz et al. [30]. The Mg-precursor flow (Cp_2_Mg) during growth was varied in steps of 30, 60 and 120 sccm (Figure 7), leading to Mg ion intensities of 252,300, 143,000 and 213,000 a.u. at the surface (Figure S12), as determined by ToF-SIMS. With the increase in the Mg-precursor flow, the bulk Mg concentration increased as well, whereas the apparent surface Mg concentration changed irregularly. This could be a consequence of some Mg segregation, which could occur at higher Mg concentrations (above 5 × 10^18^ cm^–3^). Similar conditions at the end of the growth process and subsequent cooling of the samples could also lead to similar Mg surface concentrations.

Here, we observed that the mean fluorescence intensity was highest for the 30 sccm sample as compared to other p-type surfaces. Moreover, the 30 sccm and 60 sccm functionalized p-type surfaces showed approximately similar fluorescence (Table 3 and Table S4). However, the estimated OH density was higher in the 30 sccm sample than in the other doped p-GaN surfaces (Figure S13). The hole concentration of the 30 sccm p-GaN sample, as determined by Hall measurements, was 3.5 × 10^17^ cm^–3^, whereas the other samples showed a reduced conductivity, and thus, were difficult to measure. Moreover, we observed a decrease in hole concentration and reduction in fluorescence intensity difference between blank and treated surfaces with increasing Mg concentration in p-GaN samples, similar to the observations reported in the literature [53]. Therefore, we conclude that the concentration of active Mg acceptors rather than the total Mg content plays a crucial role in the success of protein functionalization of p-GaN samples. Indeed, in heavily Mg doped samples, intrinsic lattice defects lead to a compensation of the Mg acceptors, thus reducing the expected hole concentration significantly [53] which may shift the Fermi pinning position at the surface which hypothetically leads to the misalignment with the HOMO-LUMO energy levels of the biomolecule systems, which could prevent protein surface functionalization.

Since successful surface biofunctionalization and APTES monolayer formation has taken place for p-type surfaces, we finally made an attempt to synthesize p-QW samples by introducing a Mg-precursor flow during the growth of the top capping layer. Therefore, we tried to prepare annealed p-QW samples with different capping layer thicknesses, ranging from 10 nm to 50 nm and 200 nm. Here, we observed fluorescence only on the 200 nm thick capping layer p-QW (Figure S14), which contained a thin Mg-doped top layer (Figure S15). Unfortunately, due to the delay of Mg incorporation after switching on the Mg precursor in the MOVPE process [54], thinner capping layers did not contain any reasonable Mg concentrations. However, with such a very thick capping layer, any possible sensor sensitivity vanishes, and thus, biosensing is impossible with this structure.

## 4. Conclusions

We have demonstrated that Mg doping of GaN strongly improves its bio-functionalization, which is necessary for constructing a selective biosensor platform. This is evident from the higher difference in mean fluorescence intensity between bare and protein functionalized surfaces and from XRR-based estimations of the layer thickness, while Si-doped GaN surfaces could not be bio-functionalized. However, the response of nominally undoped samples was mixed. We found minor concentrations of Mg impurities on the surface of some undoped samples, which could have promoted functionalization above some critical value. An increased Mg concentration, and thus a decreased concentration of ionizable Mg atoms in p-GaN reduced the degree of biomolecular functionalization, suggesting that an optimum dopant type and dopant content are key for the development of better biosensors.

## Figures and Tables

**Table 1:** Doping information of the samples: From the electron concentration of the n-doped samples from Hall measurements, we estimated the Si concentration, whereas both Mg and hole concentrations were measured (independently) for the Mg-doped samples using dynamic SIMS (secondary-ion mass spectrometry) and Hall measurements. Conc. in brackets means assumed concentration per cm^3^. p-GaN: p-type GaN, n-GaN: n-type GaN, u-GaN: un-doped GaN, un-doped GaN-GaInN (gallium indium nitride)-GaN quantum well structure.

Ref.	Sample	Type	SIMS Bulk Conc.		Hole and Electron Conc.
			Mg	Si	
A	Y2139	p-GaN	3.5 × 10^19^	<[1 × 10^17^]	p = 3.5 × 10^17^
B	Y2118	n-GaN	<[1 × 10^17^]	~3.3 × 10^18^	n = 3.3 × 10^18^
C	Y2151	u-GaN	<1.5 × 10^16^	<[1 × 10^17^]	<[1 × 10^17^]
D	Y2108	u-QW	<[1 × 10^17^]	<[1 × 10^17^]	<[1 × 10^17^]

**Table 2:** Mean fluorescence intensity (a.u.) of biomolecule functionalized substrates. Mean value ± standard deviations are reported, sccm: standard cubic centimeters per minute. Conc. in bracket means assumed concentration in cm^–3^.

Sample Type	Sample No.	Ref.	Carrier Conc.	Source	Blank	Treated	Difference
u-GaN	Y2092	E	<[1 × 10^17^]	unintentionally doped	60 ± 6	61 ± 7	-
u-GaN	T7066	F	<[1 × 10^17^]	unintentionally doped	40 ± 7	42 ± 6	-
u-GaN	Y2151	C	<[1 × 10^17^]	unintentionally doped	55 ± 6	70 ± 7	15
u-GaN	T9039	G	<[1 × 10^16^]	unintentionally doped	41 ± 6	62 ± 7	21
n-GaN	Y2118	B	3.3 × 10^18^	Same doping as Y2074	53 ± 4	57 ± 7	4
n-GaN	Y2072	H	7.4 × 10^17^	Si-doped	41 ± 3	41 ± 4	-
n-GaN	Y2070	I	9.1 × 10^18^	Si-doped	38 ± 5	38 ± 3	-
n-GaN	Y2073	K	2.4 × 10^17^	Si-doped	39 ± 4	41 ± 4	-
p-GaN	Y2124	L	1.5 × 10^17^	Hall (30 sccm Mg)	35 ± 5	53 ± 9	18
p-GaN	Y2139	A	3.5 × 10^17^	Hall (30 sccm Mg)	52 ± 7	67 ± 8	15
p-GaN	T5127	M	3.8 × 10^17^	Hall (30 sccm Mg)	46 ± 4	67 ± 5	21
p-GaN	T5126	N	1.6 × 10^17^	Hall (30 sccm Mg)	45 ± 4	61 ± 4	16
Glass		J			30 ± 3	48 ± 4	18
u-QW	Y2066	O	<[1 × 10^17^]	unintentionally doped	40 ± 3	41 ± 4	-
u-QW	Y2108	D	<[1 × 10^17^]	unintentionally doped	40 ± 4	44 ± 4	4
u-QW	Y1965	P	<[1 × 10^17^]	unintentionally doped	42 ± 4	42 ± 4	-
n-QW	Y2074	Q	3.3 × 10^18^	Hall	38 ± 3	43 ± 4	5
n-QW	Y2075	R	9.4 × 10^17^	Hall	38 ± 3	45 ± 4	7

**Table 3:** Mg bulk concentration, hole concentration, Mg ion intensity at the surface, mean fluorescence intensity (mean value ± standard deviation in arbitrary units; exposure—25 s; gain kept at 10 for all images) and OH density ratio of the protein functionalized substrates. The latter was estimated from the ratio of the deconvoluted O (1s) intensities in the XPS spectra corresponding to O‒H and O‒Ga species. Conc. means concentration per cm^3^.

p-GaN Cp_2_Mg Injection Level	Mg Bulk Conc.	Hole Conc.	Mg Ion Intensity (SIMS)	Fluorescence Intensity		OH Density (O-H_area_/O-Ga_area_) Ratio
				Blank	Treated	
30 sccm	5 × 10^19^	3.5 × 10^17^	252,300	46 ± 6	69 ± 9	4.7
60 sccm	9 × 10^19^	3.0 × 10^16^	143,000	49 ± 5	67 ± 8	3.5
120 sccm	2 × 10^20^	<1.0 × 10^16^	213,000	50 ± 6	52 ± 7	3.6

## Supplementary Materials

The supplemental file is available https://www.mdpi.com/1424-8220/20/15/4179/s1.

## Abbreviations

The following abbreviations are used in this manuscript:

GaN	Gallium nitride
QW	Quantum well
Stp	Streptavidin
FBR	Ferritin-Biotin-Rhodamine complex
SPB	Silane-PEG-Biotin
APTES	3-aminopropyl triethoxysilane
p-GaN	p-type GaN
n-GaN	n-type GaN
u-GaN	un-doped GaN
u-QW	un-doped GaN-GaInN-GaN quantum well structure
p-QW	GaN-GaInN-GaN quantum well structure with Mg-doped cap layer
sccm	standard cubic centimeters per minute
a.u.	arbitrary units
XRR	X-ray Reflectivity
XPS	X-ray Photoelectron Spectroscopy
MALDI-ToF	Matrix-Assisted Laser Desorption/Ionization Time-of-Flight
ToF-SIMS	Time-of-Flight Secondary Ion Mass Spectrometry
AFM	Atomic force microscopy
