# Supplementary material for: Impact of Surface Chemistry and Doping Concentrations on Biofunctionalization of GaN/Ga‒In‒N Quantum Wells
Source: Sensors (Basel). 2020 Jul 28;20(15):4179. doi: 10.3390/s20154179 (PMC7435836; doi:10.3390/s20154179)
Supplement: Supplementary file 1 [file sensors-20-04179-s001.pdf]

*sensors*

Article

# Impact of Surface Chemistry and Doping Concentrations on Biofunctionalization of GaN/Ga–In–N Quantum Wells

**Nilanjon Naskar [1,*], Martin F. Schneidereit [2], Florian Huber [3], Sabyasachi Chakrabortty [1,4], Lothar Veith [5], Markus Mezger [5], Lutz Kirste [6], Theo Fuchs [6], Thomas Diemant [7], Tanja Weil [1,5], R. Jürgen Behm [7], Klaus Thonke [3] and Ferdinand Scholz [2]**

[1] Institute of Inorganic Chemistry I, Ulm University, Albert-Einstein-Allee 11 and D-89081 Ulm, Germany; sabyasachi.c@srmap.edu.in (S.C.); Tanja.Weil@uni-ulm.de and weil@mpip-mainz.mpg.de (T.W.)
[2] Institute of Functional Nanosystems, Ulm University, Albert-Einstein-Allee 45, D-89081 Ulm, Germany; Martin.Schneidereit@uni-ulm.de (M.F.S.); Ferdinand.Scholz@uni-ulm.de (F.S.)
[3] Institute of Quantum Matter/Semiconductor Physics Group, Ulm University, Albert-Einstein-Allee 45, D-89081 Ulm, Germany; florian.huber@alumni.uni-ulm.de (F.H.); klaus.thonke@uni-ulm.de (K.T.)
[4] Department of Chemistry, SRM University AP Andhra Pradesh, Andhra Pradesh 522502, India
[5] Max Planck Institute for Polymer Research, Ackermannweg 10, D-55128 Mainz, Germany; veith@mpip-mainz.mpg.de (L.V.); mezger@mpip-mainz.mpg.de (M.M.)
[6] Fraunhofer Institute for Applied Solid State Physics, Tullastrasse 72, D-79108 Freiburg, Germany; Lutz.Kirste@iaf.fraunhofer.de (L.K.); Theodor.Fuchs@iaf.fraunhofer.de (T.F.)
[7] Institute of Surface Chemistry and Catalysis, Ulm University, Albert-Einstein-Allee 47, D-89081 Ulm, Germany; thomas.diemant@uni-ulm.de (T.D.); juergen.behm@uni-ulm.de (R.J.B.);
* Corresponding Author: nilanjon.naskar@uni-ulm.de

Received: 24 June 2020; Accepted: 23 June 2020; Published: date

**Supporting Information**

**Figure S1**. XPS characterization of blank and treated surfaces.

**Figure S2**. Surface images from ToF-SIMS measurements.

**Figure S3**. Fluorescence images of biomolecular functionalized surfaces.

**Figure S4**. Additional XPS characterizations.

**Figure S5**. X-ray reflectivity pattern of APTES functionalized p-GaN surfaces.

**Figure S6**. Additional x-ray reflectivity results.

**Figure S7.** X-ray reflectivity pattern of all APTES functionalized surfaces.

**Figure S8**. Additional fluorescence characterizations of bio-functionalized surfaces.

**Figure S9**. ToF-SIMS measurements of u-GaN surfaces.

**Figure S10**. AFM topographic images of biomolecular functionalized p-GaN surfaces.

**Figure S11**. Fluorescence images of bio-functionalized n-GaN surfaces with varying doping level.

**Figure S12**. ToF-SIMS measurements of p-GaN surfaces of varying doping level.

**Figure S13**. XPS and fluorescence characterizations of bio-functionalized p-GaN surfaces of varying doping level.

**Figure S14**. Fluorescence images of bio-functionalized p-QW.

**Figure S15**. Additional ToF-SIMS characterizations.

**Figure S16**. Adhesion mapping of APTES-coated surfaces.

**Figures S17–S20**. Additional fluorescence images of biofunctionalized samples

**Figures S21, S22**. Additional contact angle measurements.

**Figure S23**. Additional AFM images of biofunctionalized p-GaN samples.

**Tables S1–2**. X-ray reflectivity characterizations of APTES coated p-GaN surfaces.

**Table S3**. Ellipsometry results for blank and APTES coated GaN surfaces.

**Table S4**. Fluorescence mean intensity of bio-functionalized p-GaN surfaces.

**References**

**(a)**

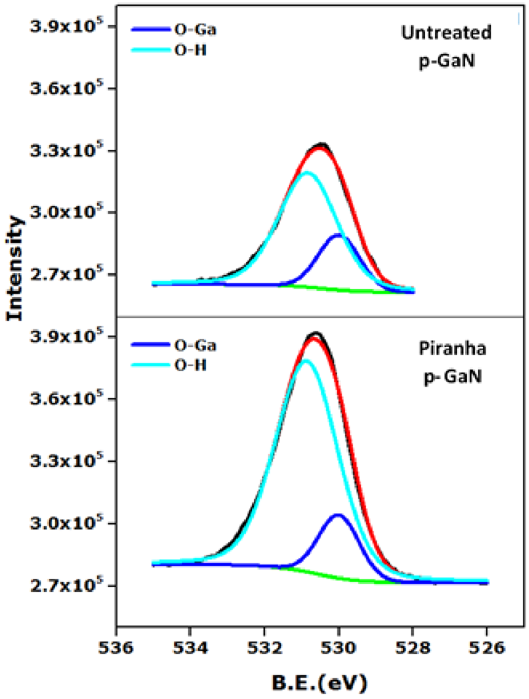


| Untreated p-GaN | B.E. (eV) | Area | $Area_{O-H}/Area_{O-Ga}$ |
|---|---|---|---|
| O-Ga | 530 | 36982 | 3.3 |
| O-H | 530.8 | 122516 | |

| Piranha treated p-GaN | B.E. (eV) | Area | $Area_{O-H}/Area_{O-Ga}$ |
|---|---|---|---|
| O-Ga | 530 | 41654 | 5.7 |
| O-H | 530.9 | 236226 | |

**(b)**

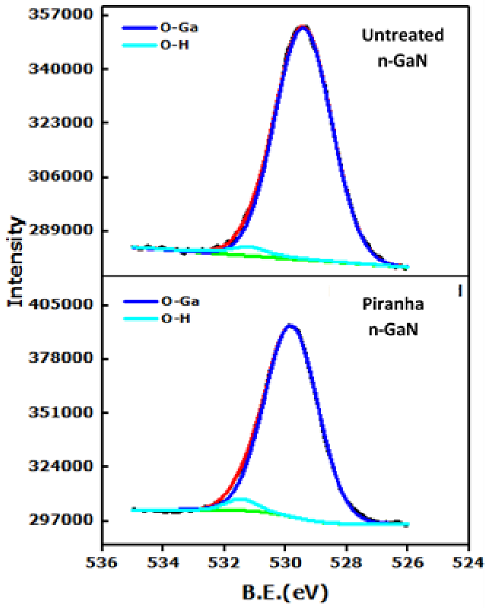


| Untreated n-GaN | B.E. (eV) | Area | $Area_{O-H}/Area_{O-Ga}$ |
|---|---|---|---|
| O-Ga | 529.4 | 169866 | 0.04 |
| O-H | 531.2 | 6725 | |

| Piranha treated n-GaN | B.E. (eV) | Area | $Area_{O-H}/Area_{O-Ga}$ |
|---|---|---|---|
| O-Ga | 529.8 | 211101 | 0.03 |
| O-H | 531.4 | 7202 | |

**Figure 1.** O1s XPS spectra of blank and piranha treated (20 min) (**a**) p-GaN, (**b**) n-GaN surfaces and their respective fitting parameters for the deconvoluted curves.

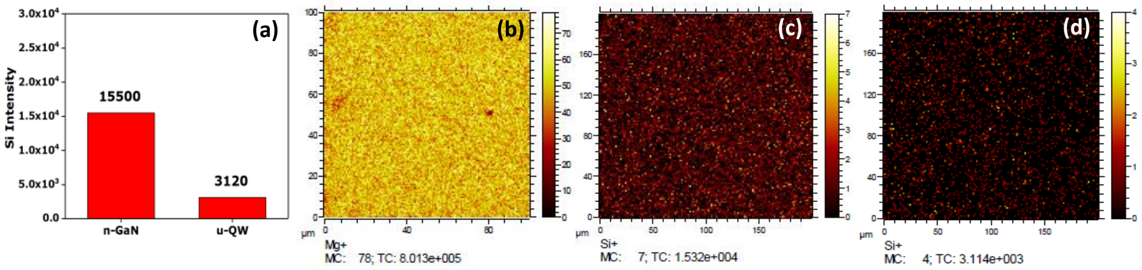


**Figure S2.** ToF-SIMS measurements: (**a**) relative Si ion intensity comparison between bare n-GaN and u-QW surfaces. Surface images showing signal distribution of (**b**) $Mg^+$ ions in p-GaN, (**c**) $Si^+$ ions in n-GaN, (**d**) $Si^+$ ions in QW.

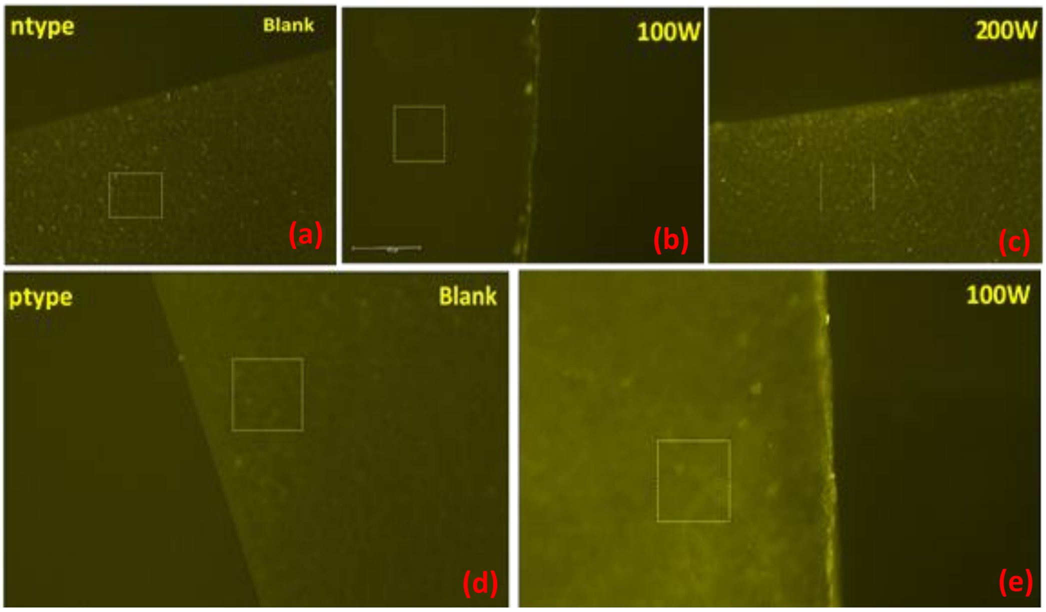


**Figure S3.** Fluorescence micrographs of (**a**) blank n-GaN, (**b**) 100 W at 20 min, and (**c**) 200 W at 10 min $O_2$ plasma treated biomolecular functionalized n-GaN surfaces. (**d**) Blank p-GaN, (**e**) 100 W at 20 min $O_2$ plasma treated bio-functionalized p-GaN surface.

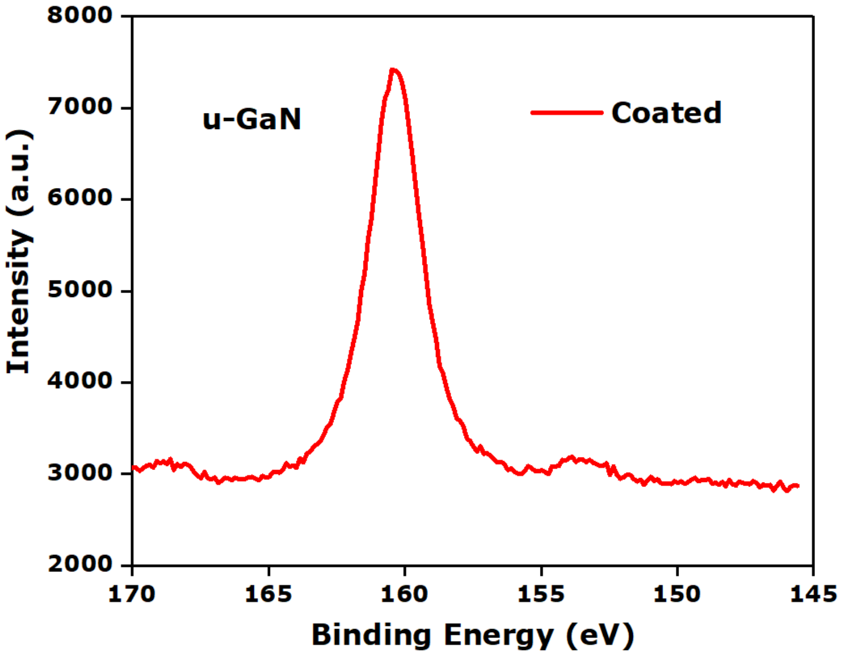


**Figure S4.** Si 2s XPS spectrum of APTES functionalized undoped GaN (u-GaN, Y2151).

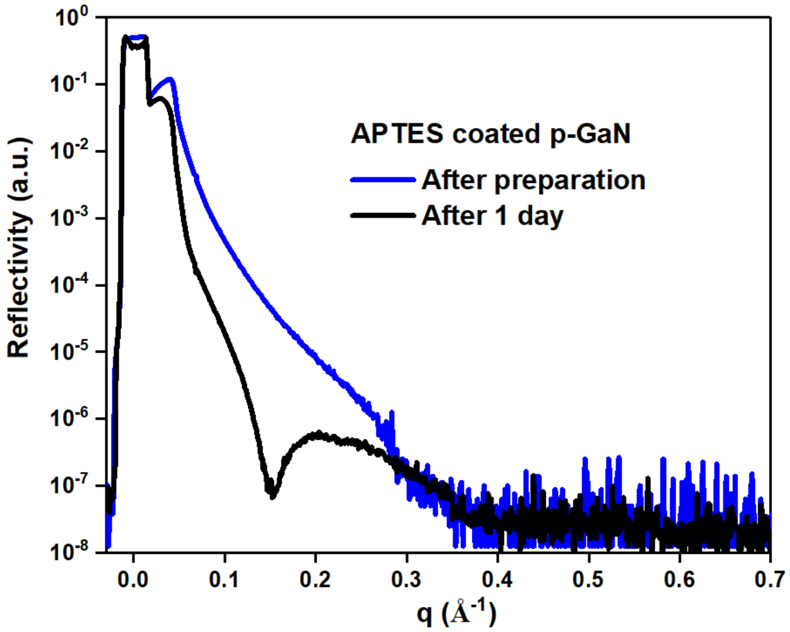


**Figure S5.** XRR pattern of APTES functionalized p-GaN after preparation (blue) and after 1 day (black).

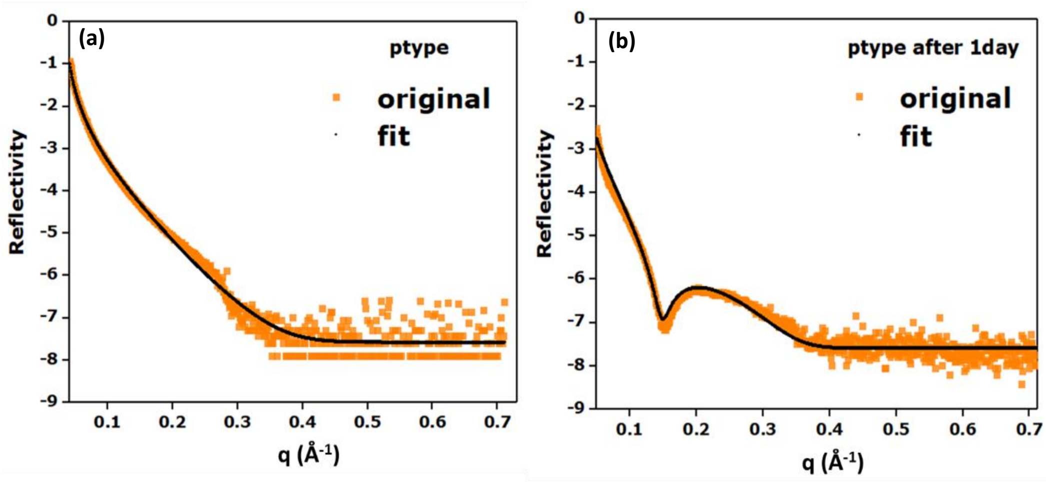


**Figure S6.** Fitting of XRR pattern into 2-layer model for APTES functionalized p-GaN (**a**) after preparation, (**b**) after 1 day [1].

**Table S1.** The fitted parameters and oxide layer thickness on APTES coated p-GaN (original). Chi$^2$: 0.085. For bare and APTES coated p-GaN, the AFM surface roughness and maximum roughness height was estimated to be 5 Å and 6.7 Å, respectively. SLD means scattered length density. Backing refers to the substrate. The SLD, thickness and roughness values were obtained from Motofit simulation. APTES film density was calculated according to the procedure mentioned by Kumar et al. [2].

| Layer on p-GaN | Thickness (Å) | SLD ($10^{-6}$ Å) | Film Density (g/cm$^3$) | Roughness (Å) |
|---|---|---|---|---|
| APTES | 8.3 | 5.3 | 0.56 | 6.0 |
| Native oxide (GaO) | 3.6 | 7.0 | | 4.4 |
| Backing | - | 29.4 | | 5.8 |

**Table S2.** The fitted parameters for increased layer thickness on p-GaN surface (in the following day after surface preparation). Chi$^2$: 0.049.

| Layer on p-GaN | Thickness (Å) | SLD ($10^{-6}$ Å) | Roughness (Å) |
|---|---|---|---|
| APTES | 18.2 | 6.2 | 5.0 |
| Native oxide (GaO) | 3.6 | 7.0 | 4.4 |
| Backing | - | 13.9 | 5.6 |

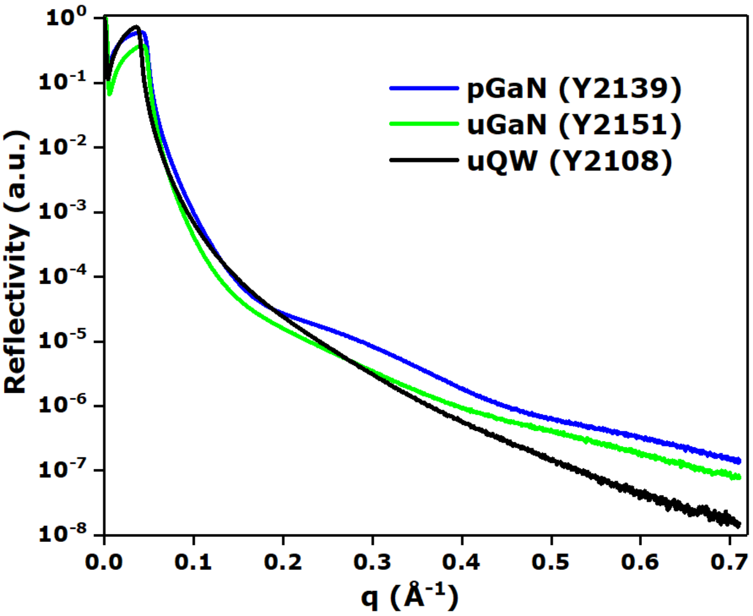


**Figure S7.** XRR pattern of APTES functionalized p-GaN (blue, relative humidity 40–38%), u-GaN (green, relative humidity 37–39%) and u-QW (red, relative humidity 36-35%). The APTES layer thicknesses on p-GaN and u-GaN are estimated to be 9.5 nm and 9.2 nm. During the XRR measurements, the environmental conditions were kept at 19 ± 1 °C air temperature and 37 ± 3% relative humidity.

**Table S3.** The Ellipsometric parameters of blank and APTES coated GaN surfaces. Ψ is ellipsometric parameter measured at 5 eV at 70°. EMA is effective medium approximation according to Bruggeman – that may be needed due to surface roughness or contaminants [3–5]. Conc. means concentration in $cm^{-3}$ and the brackets denotes assumed values. Large differences in GaN thickness affect the reliability of fitting of theoretical model. The EMA layer thickness increases with APTES treatment, therefore the presence of APTES or the increase in surface roughness due to the measurement process can be plausible reasons. The MOVPE data showed the expected AlN layer thickness for all the samples to be around 20nm. The expected GaN layer thickness of the samples are ~1.2 μm (Y2139), ~1.0 μm (Y2151), ~1.1 μm (Y2072) and ~2.2 μm (Y2118). The layer thickness expected from MOVPE matches quite closely to that obtained from ellipsometric measurements.

| Sample | Carrier Conc. | Type | Thickness AlN (nm) | Thickness GaN (nm) | Thickness EMA (nm) | Ψ (degree) |
|---|---|---|---|---|---|---|
| p-GaN (A) | $3.5 \times 10^{17}$ | Blank | 21.4 | 1368 | 1.9 | 6.365 |
| | | Coated | 21.5 | 1367 | 2.3 | 6.709 |
| u-GaN (C) | $1.0 \times 10^{16}$ | Blank | 22.0 | 1036 | 1.7 | 6.235 |
| | | Coated | 22.9 | 1016 | 2.8 | 7.267 |
| n-GaN (B) | $3.3 \times 10^{18}$ | Blank | 30.6 | 2192 | 2.0 | 6.467 |
| | | Coated | 32.2 | 2184 | 2.1 | 6.506 |
| n-GaN (H) | $7.7 \times 10^{17}$ | Blank | 19.2 | 1125 | 1.9 | 6.324 |
| | | Coated | 28.9 | 1090 | 2.1 | 6.573 |

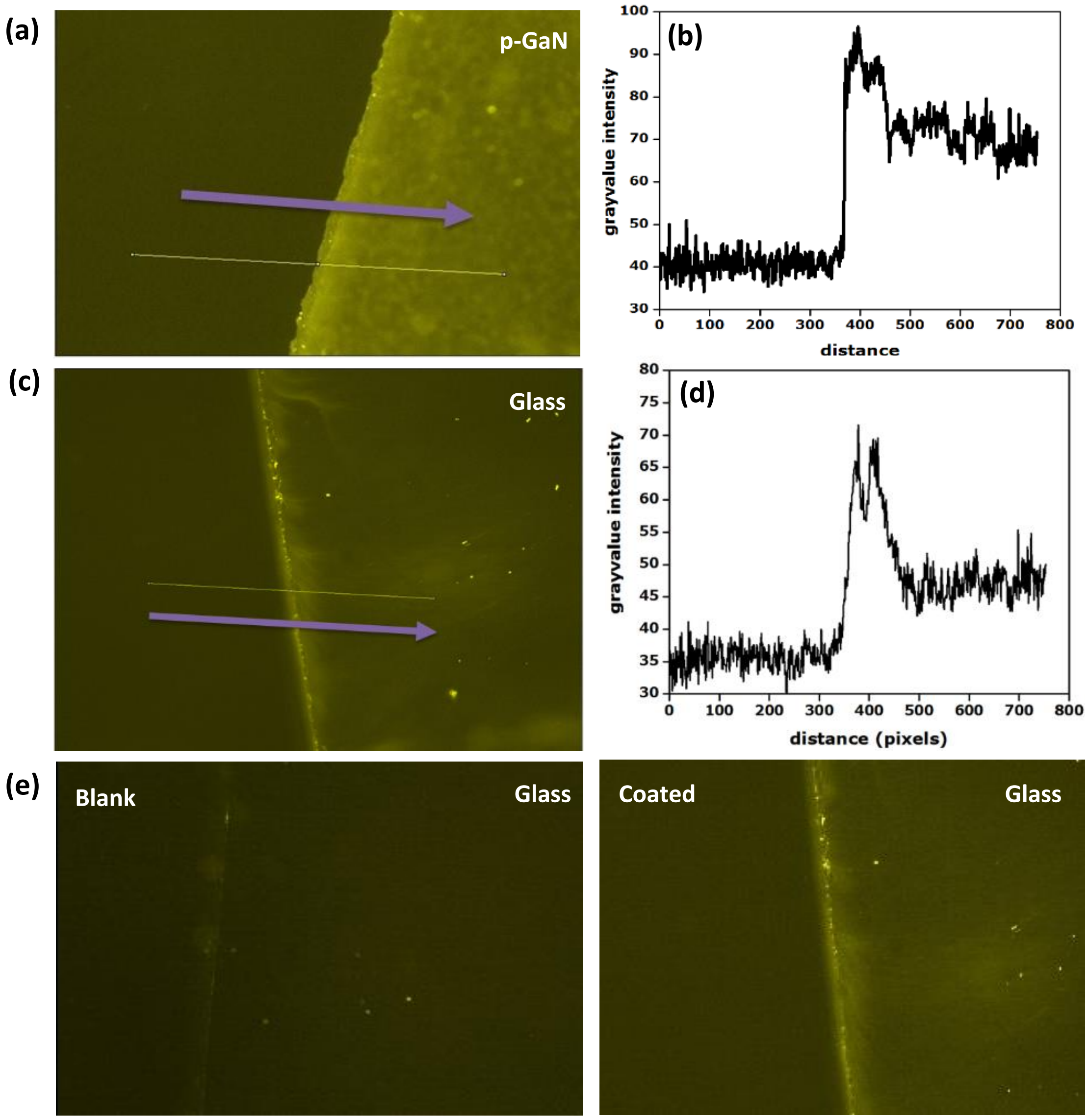


**Figure S8.** (**a**) Fluorescence images of bio-functionalized p-GaN, (**b**) line profile of grey-value intensity on treated p-GaN, (**c**) fluorescence images of bio-functionalized glass, (**d**) line profile of grey-value intensity on treated glass, (**e**) fluorescence micrographs of blank and bio-functionalized glass. Due to the uneven edge boundary, reflection of light makes the edge brighter than rest of the areas.

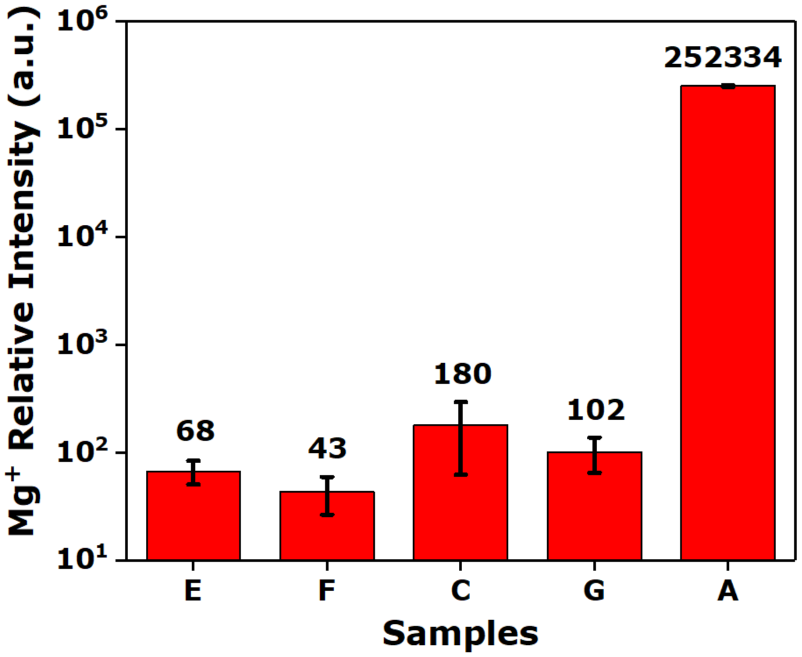


**Figure S9.** Relative $Mg^+$ intensity (in logarithmic scale) from the ToF-SIMS measurements (mean ± SE) in blank undoped GaN (**E**, **F**, **C** and **G**) and p-GaN (**A**) samples. Three different spots on the same substrate were measured.

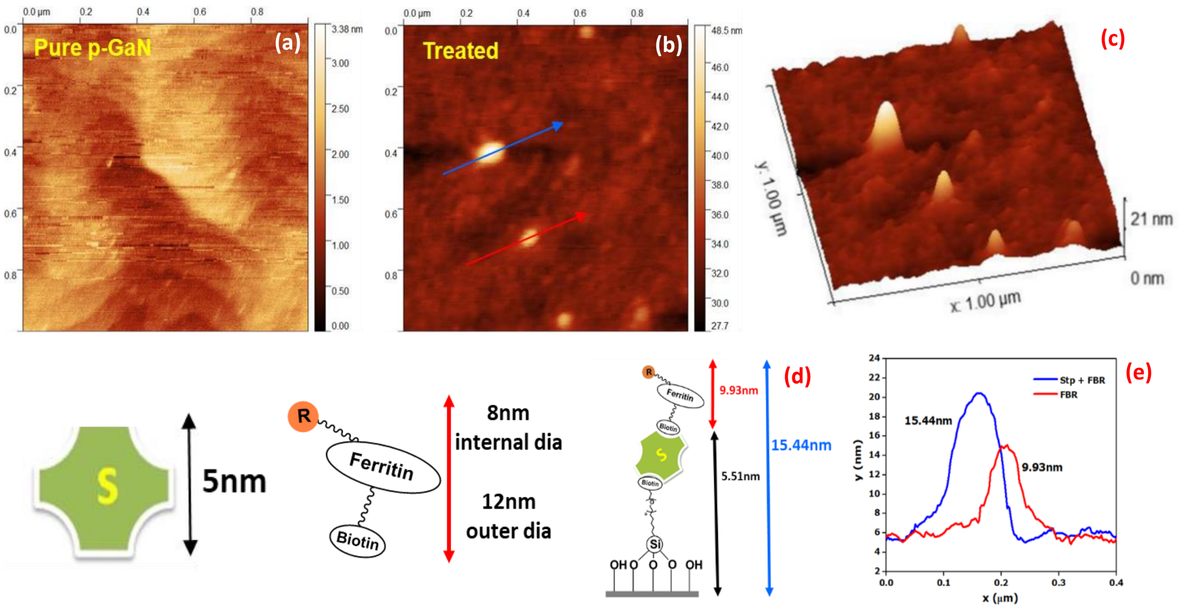


**Figure S10.** AFM characterization of (**a**) blank, (**b**) bio-functionalized p-GaN, (**c**) 3D view at 1 µm x 1 µm scale. (**d**) Size description of different biomolecules, (**e**) height profile of marked features [6].

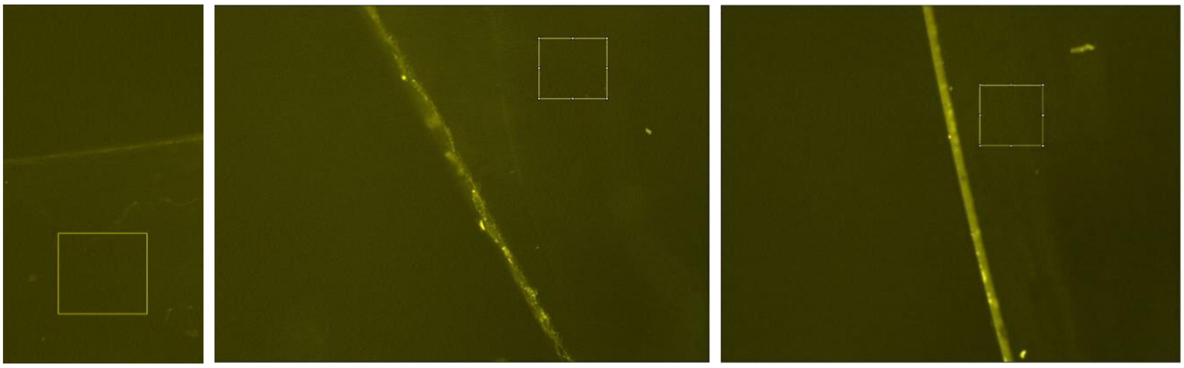

**Figure S11.** Fluorescence images of biomolecular functionalized n-GaN with different Si doping levels: (**a**) $<10^{17}$ (nominally undoped), (**b**) $2 \times 10^{17}$ (nominally undoped), and (**c**) $9 \times 10^{18}$ $cm^{-3}$. Due to the uneven edge boundary, light reflection creates a brighter edge relative to other areas.

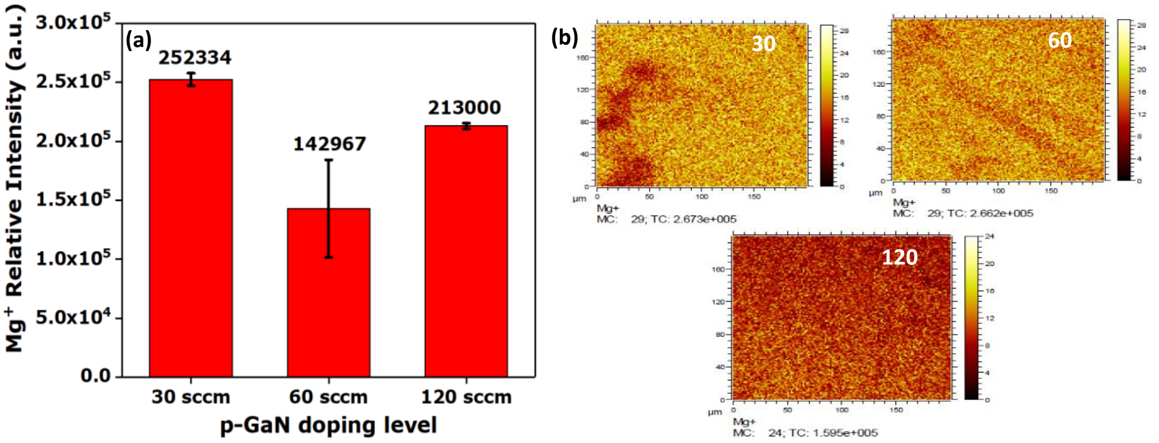


**Figure S12.** Variation of Mg ion intensity in (**a**) differently doped, blank p-GaN; (**b**) surface images of Mg ion signal distribution of differently doped blank p-GaN surfaces from ToF-SIMS measurements.

The difference in fluorescence mean intensity between edges of bio-functionalized p-type substrate was closely similar in 30 sccm doped p-GaN, indicating the formation of a homogeneous biomolecular layer. In other substrates, the fluorescence intensity difference was significantly larger and thus inhomogeneous biomolecular layer can be expected to form on other surfaces.

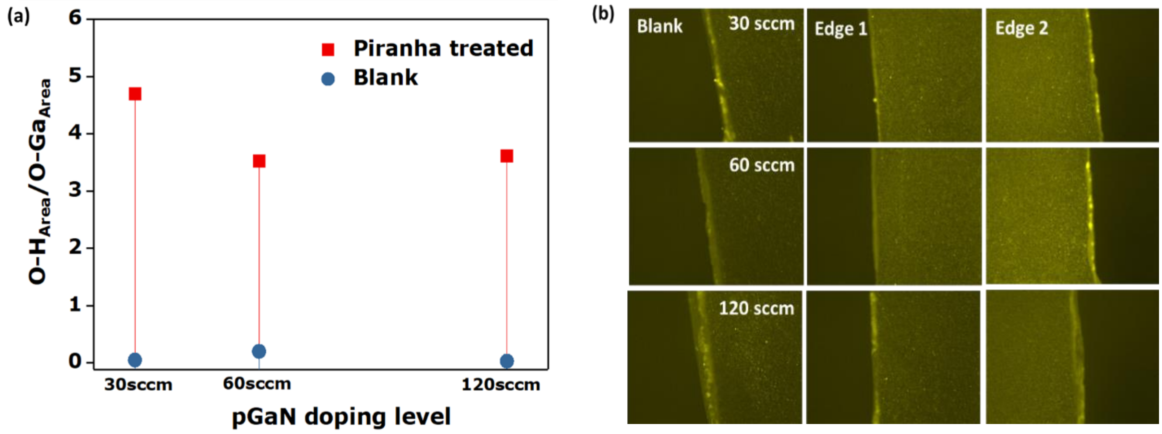


**Figure S13.** (**a**) OH density variation (from XPS) w.r.t Mg doping level in p-GaN; (**b**) fluorescence micrographs of near edges bio-functionalized p-GaN surfaces with various Mg doping levels.

**Table S4.** Fluorescence mean intensity of biomolecular functionalized differently doped p-GaN surfaces.

| Samples | Hole concentration ($cm^{-3}$) | Mean Intensity (a.u.) | | |
|---|---|---|---|---|
| | | Blank | Coated Side | |
| | | | Edge 1 | Edge 2 |
| 30 sccm | $3.5 \times 10^{17}$ | 44 ± 6 | 60 ± 8 | 70 ± 7 |
| 60 sccm | $3.0 \times 10^{16}$ | 41 ± 6 | 54 ± 7 | 73 ± 9 |
| 120 sccm | $1.0 \times 10^{16}$ | 41 ± 5 | 45 ± 6 | 57 ± 5 |

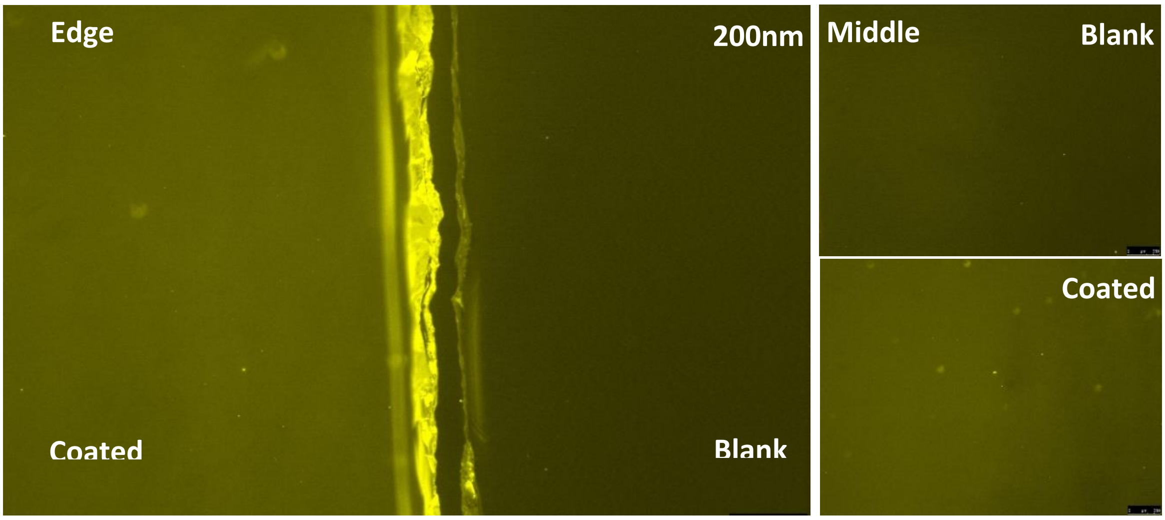


**Figure S14.** Fluorescence micrographs of bio-functionalized p-QW of 200 nm top cap layer thickness. Images were taken at an exposure time of 25 s and gain 10.

ToF-SIMS results of 200 nm, 50 nm annealed and unannealed cap layer p-doped QW showed significant presence of Mg dopant in 200 nm thick cap layer, but very low trace amount of Mg level was detected in 50 nm thick cap layer p-doped QWs. Low Mg level might be a probable reason for the unsuccessful protein functionalization, and thus no fluorescence was observed in 50 nm thick cap layer based p-doped QWs.

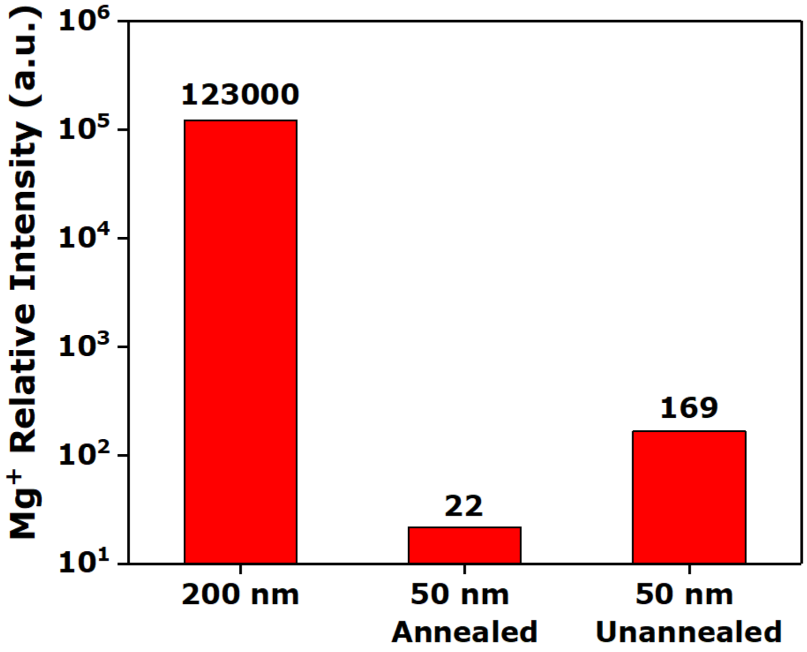


**Figure S15.** Variation of Mg ion intensity (in logarithmic scale) in p-QWs with a cap layer thickness of 200 nm and 50 nm thick cap layer, the latter before and after annealing from ToF-SIMS measurements.

We performed additional AFM measurements to identify the presence of APTES coating on n-GaN and p-GaN surfaces from adhesion mapping measurements. The adhesion force of APTES coated n-GaN (13.1 nN ± 400 pN) was less than that of APTES coated p-GaN (17.2 nN ± 180 pN) and glass (18.4 nN ± 300 pN). It is known that the presence of biomolecules can make the surface very sticky in nature, thus high adhesion force can be expected. The high adhesion force values indicate that the APTES functionalization was successful on p-GaN and glass, and not on n-GaN.

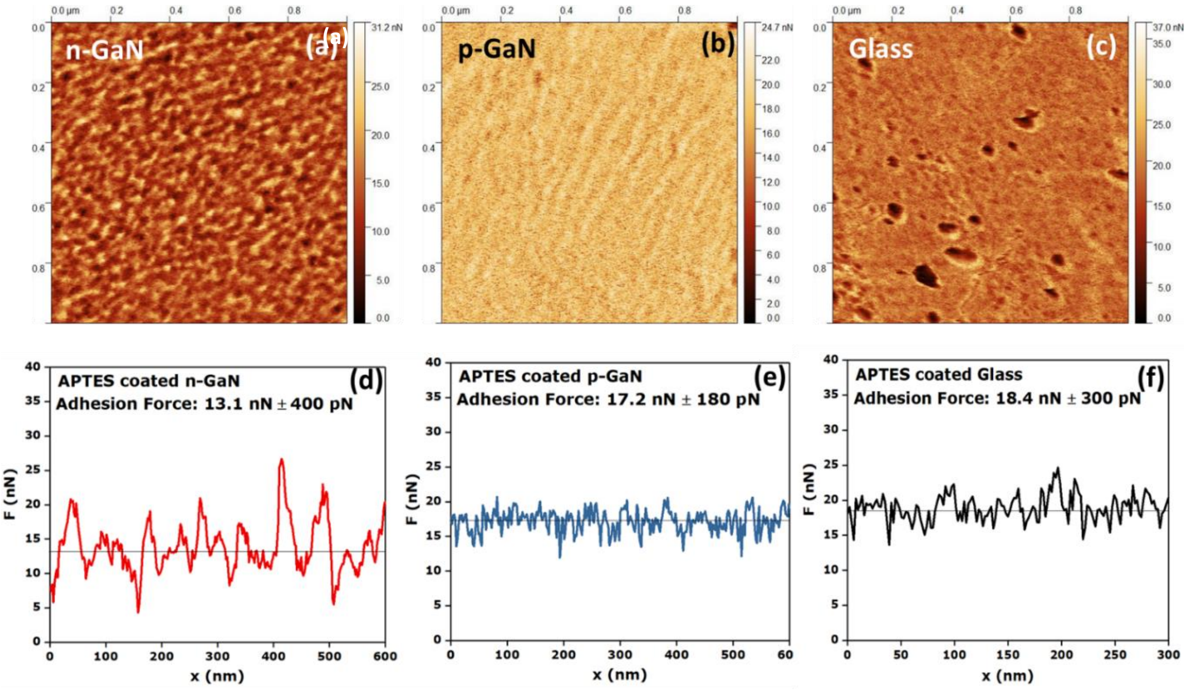


**Figure S16.** Adhesion maps of APTES functionalized (**a**) n-GaN (**B**), (**b**) p-GaN (**A**), (**c**) glass (**J**). Adhesion force profiles for APTES functionalized (**d**) n-GaN (**B**), (**e**) p-GaN (**A**) and (f) glass (**J**).

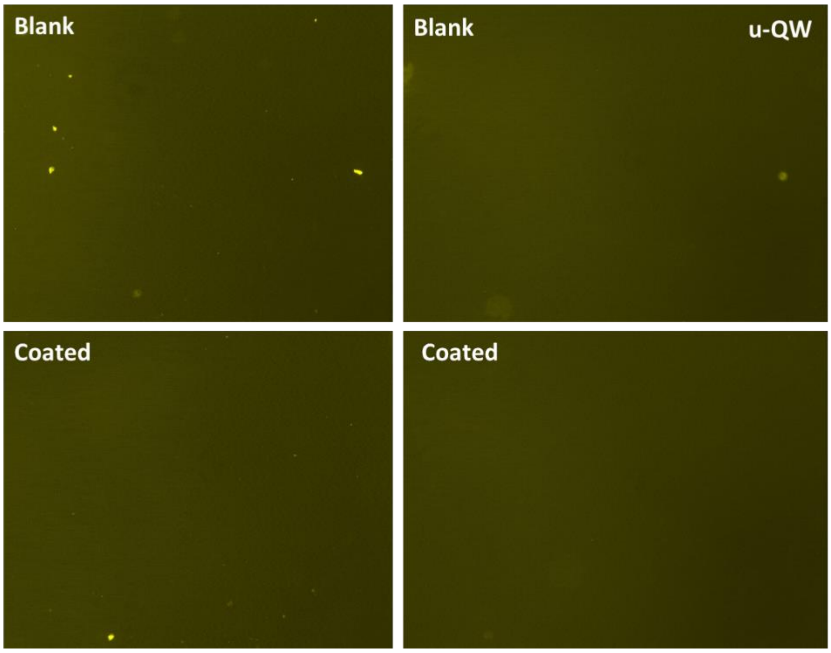


**Figure S17.** Fluorescence images of biomolecule-functionalized undoped QW (u-QW) samples.

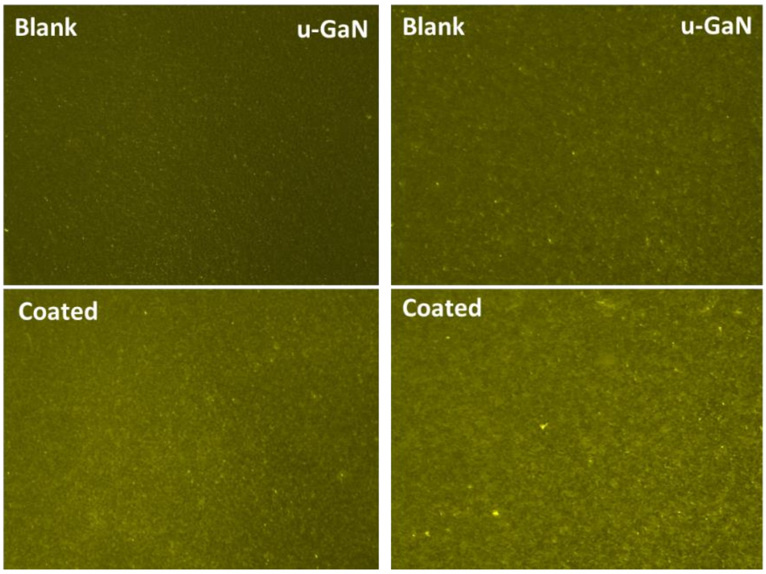


**Figure S18.** Fluorescence images of biomolecule-functionalized undoped GaN (u-GaN) samples.

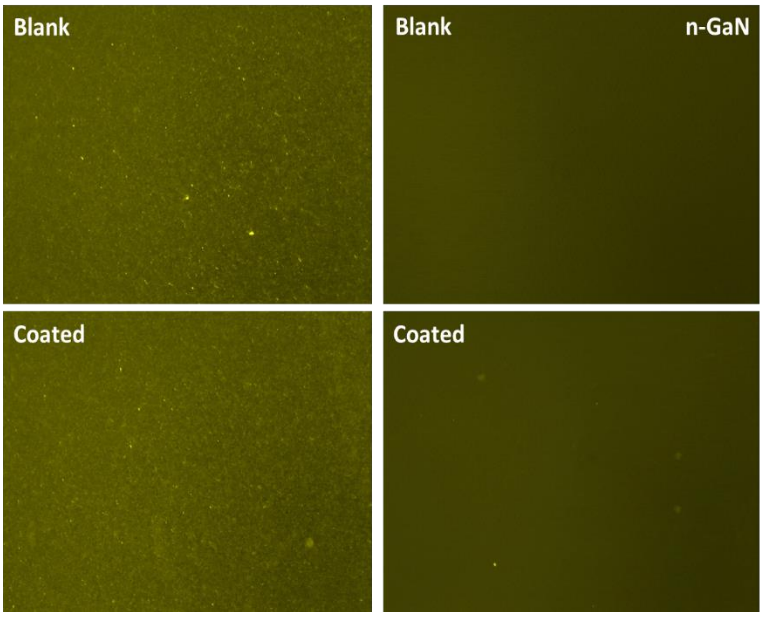


**Figure S19.** Fluorescence images of biomolecule-functionalized Si–doped GaN (n-GaN) samples.

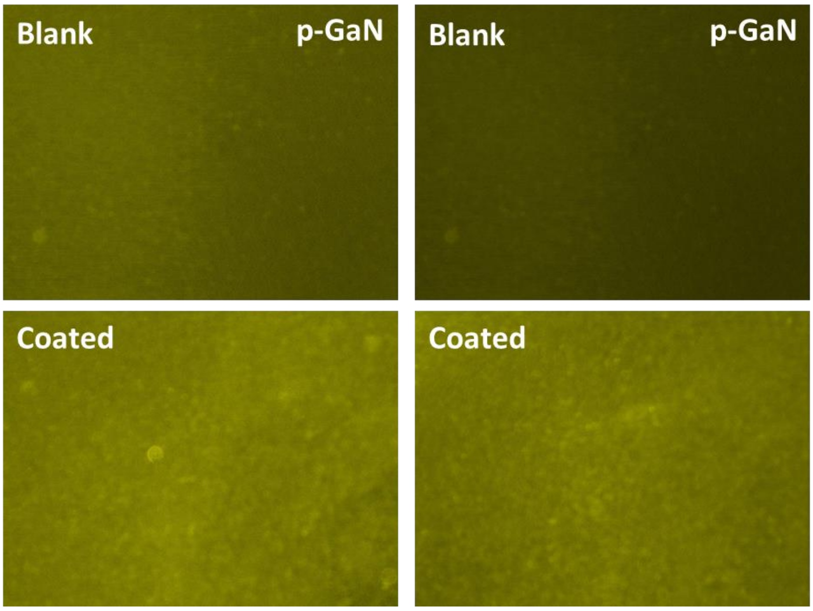


**Figure S20.** Fluorescence images of biomolecule-functionalized Mg–doped GaN (p-GaN) samples.

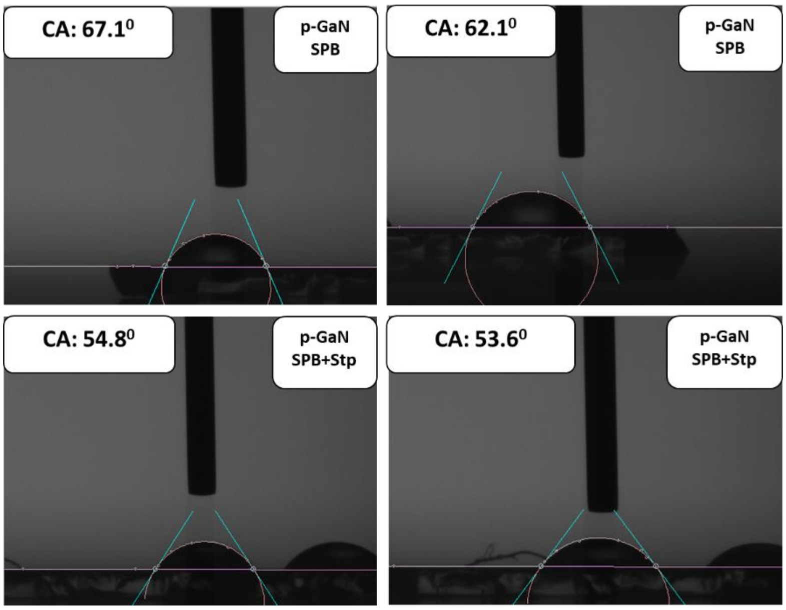


**Figure S21.** Contact angle of water drops on bare and silane-PEG-biotin (SPB) coated p-GaN (**A**) surfaces with and without streptavidin (Stp).

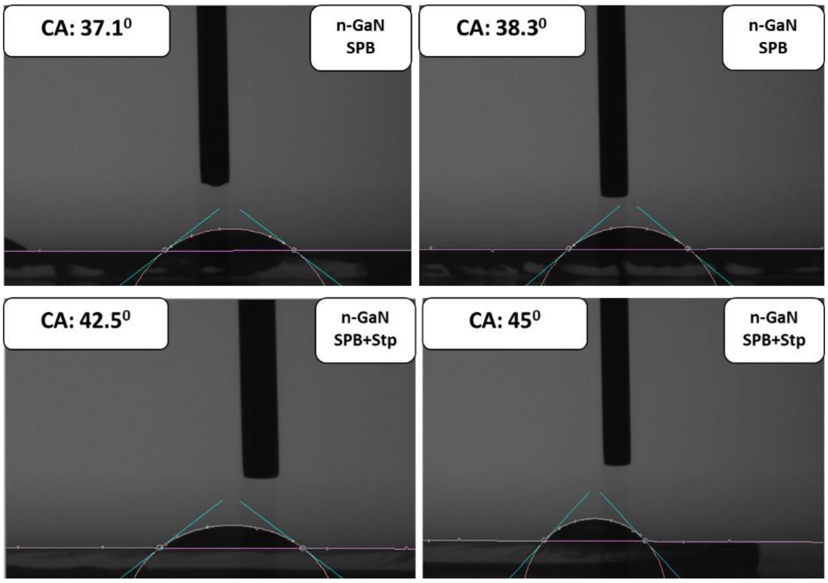


**Figure S22.** Contact angle of water drops on bare and silane-PEG-biotin (SPB) coated n-GaN (**B**) surfaces with and without streptavidin (Stp).

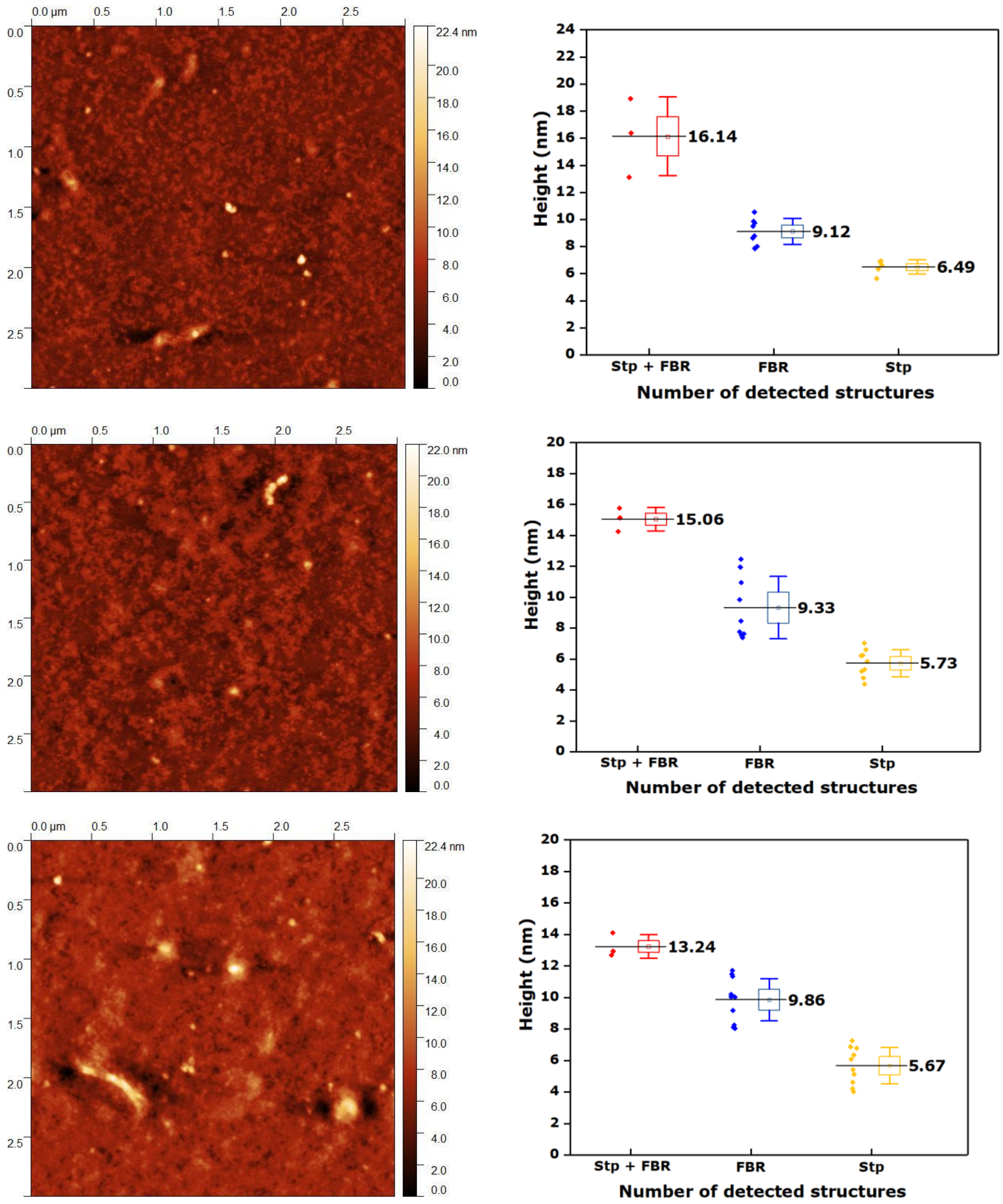


**Figure S23.** AFM images of three protein functionalized p-GaN (**A**) samples and their respective height distribution. Nanostructures were categorized according to their dimensions and three different features were recognized with heights of about 5–7 nm, 9–10 nm and 13–16 nm, which can be ascribed to Stp (5 nm), FBR complexes (10 nm) and Stp bound FBR complexes (15 nm).

## References

1. Argekar, S.U.; Kirley, T.L.; Schaefer, D.W. Determination of structure-property relationships for 3-aminopropyltriethoxysilane films using x-ray reflectivity. *J. Mater. Res.* **2013**, *28*, 1118–1128, doi:10.1557/jmr.2013.54.
2. Kumar, P.; Gupta, M.; Deshpande, U.P.; Phase, D.M.; Ganesan, V.; Stahn, J. Density and Microstructure of Amorphous Carbon Thin Films. *arXiv* **2018,** arXiv180101739 Cond-Mat.
3. Bruggeman, D.A.G. Berechnung verschiedener physikalischer Konstanten von heterogenen Substanzen. I. Dielektrizitätskonstanten und Leitfähigkeiten der Mischkörper aus isotropen Substanzen. *Ann. Phys.* **1935**, *416*, 636–664, doi:10.1002/andp.19354160705.
4. Kostruba, A.; Stetsyshyn, Y.; Mayevska, S.; Yakovlev, M.; Vankevych, P.; Nastishin, Y.; Kravets, V. Composition, thickness and properties of grafted copolymer brush coatings determined by ellipsometry: calculation and prediction. *Soft Matter* **2018**, *14*, 1016–1025, doi:10.1039/C7SM02285A.
5. Williams, E.H.; Davydov, A.V.; Motayed, A.; Sundaresan, S.G.; Bocchini, P.; Richter, L.J.; Stan, G.; Steffens, K.; Zangmeister, R.; Schreifels, J.A.; et al. Immobilization of streptavidin on 4H–SiC for biosensor development. *Appl. Surf. Sci.* **2012**, *258*, 6056–6063, doi:10.1016/j.apsusc.2012.02.137.
6. Hynninen, V.; Vuori, L.; Hannula, M.; Tapio, K.; Lahtonen, K.; Isoniemi, T.; Lehtonen, E.; Hirsimäki, M.; Toppari, J.J.; Valden, M.; et al. Improved antifouling properties and selective biofunctionalization of stainless steel by employing heterobifunctional silane-polyethylene glycol overlayers and avidin-biotin technology. *Sci. Rep.* **2016**, *6*, 29324, doi:10.1038/srep29324.
